# The Role of Microbial Community Composition in Controlling Soil Respiration Responses to Temperature

**DOI:** 10.1371/journal.pone.0165448

**Published:** 2016-10-31

**Authors:** Marc D. Auffret, Kristiina Karhu, Amit Khachane, Jennifer A. J. Dungait, Fiona Fraser, David W. Hopkins, Philip A. Wookey, Brajesh K. Singh, Thomas E. Freitag, Iain P. Hartley, James I. Prosser

**Affiliations:** 1 University of Aberdeen, Cruickshank Building St Machar Drive, Aberdeen AB24 3UU, United Kingdom; 2 University of Exeter Amory Building, Rennes Drive, Exeter EX4 4RJ, United Kingdom; 3 Hawkesbury Institute for the Environment, University of Western Sydney, Penrith 2751 NSW, Australia; 4 Sustainable Soils and Grassland Systems Department, Rothamsted Research North Wyke, Okehampton, EX20 2SB, United Kingdom; 5 School of Energy, Environment and Agrifood, Cranfield University, Cranfield, Bedfordshire, MK43 0AL, United Kingdom; 6 The Royal Agricultural University, Cirencester, Gloucestershire, GL7 6JS, United Kingdom; 7 Institute of Life & Earth Sciences, Heriot-Watt University, Edinburgh, EH14 4AS, United Kingdom; 8 The James Hutton Institute, Craigiebuckler, Aberdeen AB15 8QH, United Kingdom; Leibniz-Institute of Vegetable and Ornamental Crops, GERMANY

## Abstract

Rising global temperatures may increase the rates of soil organic matter decomposition by heterotrophic microorganisms, potentially accelerating climate change further by releasing additional carbon dioxide (CO_2_) to the atmosphere. However, the possibility that microbial community responses to prolonged warming may modify the temperature sensitivity of soil respiration creates large uncertainty in the strength of this positive feedback. Both compensatory responses (decreasing temperature sensitivity of soil respiration in the long-term) and enhancing responses (increasing temperature sensitivity) have been reported, but the mechanisms underlying these responses are poorly understood. In this study, microbial biomass, community structure and the activities of dehydrogenase and *β-*glucosidase enzymes were determined for 18 soils that had previously demonstrated either no response or varying magnitude of enhancing or compensatory responses of temperature sensitivity of heterotrophic microbial respiration to prolonged cooling. The soil cooling approach, in contrast to warming experiments, discriminates between microbial community responses and the consequences of substrate depletion, by minimising changes in substrate availability. The initial microbial community composition, determined by molecular analysis of soils showing contrasting respiration responses to cooling, provided evidence that the magnitude of enhancing responses was partly related to microbial community composition. There was also evidence that higher relative abundance of saprophytic *Basidiomycota* may explain the compensatory response observed in one soil, but neither microbial biomass nor enzymatic capacity were significantly affected by cooling. Our findings emphasise the key importance of soil microbial community responses for feedbacks to global change, but also highlight important areas where our understanding remains limited.

## Introduction

Soils contain a massive stock of terrestrial carbon (C) [1, 2], estimated at approximately 2500 Pg C, much of which is considered vulnerable to climatic warming [3, 4]. Respiration releases approximately 119 Pg C annually from the land surface, ~50% of which is due to microbial activity [5, 6]. In the short term, soil microbial respiration increases approximately exponentially with increasing temperature over the typical range of soil temperatures [7] and a positive correlation between soil respiration and temperature has been observed in many field and laboratory studies [4, 8, 9]. This has prompted the view that rising global temperatures will increase the respiration rates of microorganisms that decompose soil organic carbon (SOC) [10–12]; increased CO_2_ emissions through enhanced SOC decomposition have the potential to increase climate forcing by up to 40% [13, 14].

However, in long-term warming studies the initial stimulation of soil respiration often declines over time [8, 15, 16]. This can be explained partly by loss of the most readily-decomposable SOC pool, but there have also been suggestions that microbial communities respond to warming in such a way as to compensate for the increase in soil temperature, promoting a gradual reduction in respiration rates in warmed soils [17–20]. Mechanisms behind such responses could include physiological responses of individual microbial phylotypes, genetic changes within species (adaptation) and ecological responses associated with change in community composition.

Karhu *et al*. [21] used a soil cooling approach [22] to investigate the potential for compensatory community level responses to warming in a range of soils from different climates and ecosystem types. This study investigates the same soil samples incubated by Karhu *et al*. [21] but comparing only the cooling treatment to the control.

Enhancing microbial community responses (responses that increased the mid- to long-term (90 days) effects of the temperature changes on rates of respiration after cooling) were found to be much more common than compensatory responses (responses that decreased the effects of the temperature changes on rates of respiration after cooling). This was especially true for high-latitude soils. Therefore, it explains the unbalanced soil distribution grouped by soil respiration responses in this study and also the limitations to complete some statistical analyses. Critically, although enhancing responses were more common than compensatory responses, the full range of potential responses was observed and it is important to understand the mechanisms underlying these contrasting responses.

The aim of this study was to investigate whether initial microbial community composition and/or shifts in community composition in response to temperature change could explain the three different microbial respiration responses (enhancing, compensatory and no-response) observed by Karhu *et al*. [21], testing the following hypotheses. Firstly, we suggested that the dominance of enhancing responses after cooling is caused by changes in community composition overshadowing potential acclimation or adaptive responses within individuals and populations. This hypothesis predicts greater changes in community composition in soils exhibiting enhancing responses than in soils exhibiting either no-response or compensatory responses, particularly for soils sampled at high latitude, where low temperatures may apply the greatest selection pressure [23], despite a general decrease in microbial biomass turnover at low temperatures. In contrast, for compensatory response, a limited number of changes in microbial community composition were expected after cooling.

Secondly, Karhu *et al*. [21] found that the reduction in respiration rates in cooled soils showing enhancing responses increased over time relative to controls. This may be related to a reduction in the ability of microbes to break down low quality/recalcitrant organic matter, whose decomposition has been shown to be highly temperature sensitive [24, 25]. Fungi and some bacterial species play a key role in the decomposition of recalcitrant organic matter and, if enhancing responses are associated with development of a microbial community decomposing a reduced diversity of substrates, we hypothesised that the abundance of diverse microbial populations degrading recalcitrant compounds will decline. In contrast, the abundance of microbial populations degrading recalcitrant organic matter will increase in cooled soil exhibiting compensatory response [26, 27].

Finally, we investigated changes in total microbial biomass, biomass-specific respiration in substrate-unlimited conditions and dehydrogenase and *β-*glucosidase activities, which have been suggested as explanations for the compensatory responses previously observed in monocultures, soils under substrate-unlimited conditions, and in models incorporating microbial community dynamics [28–31].

## Materials and Methods

### Soil properties

Intact topsoil cores (100 mm diameter x 100 mm depth) were taken from different ecosystems and climatic regions (20 cores per site) and transferred to the University of Exeter at temperatures similar to that of their origin [21]. The permission to collect soil samples for each location was sought from the appropriate authority (see Table 1). Soil properties including pH and water, C and N contents were characterized as described in Karhu *et al*. [21] and each soil was identified by geographic/climatic area including 1 (Sweden/subarctic), 2 (Scotland/cool temperate through to sub-alpine), 3 (England/temperate), and 4 (Italy, Spain/Mediterranean) and ecosystem type: A (arable), C (coniferous evergreen forest), D (deciduous broadleaf forest), G (grassland), and H (ericaceous heath) [21].

**Table 1:** Authorities who issued the permission for soil sampling.

Site	MAT	Authority who issued the permission	Contact at the sampling time
1A	5.1	Swedish Agricultural University	Goran Bergkvist
1C	2.8	Umea University	Reiner Giesler
1D	-2.0	Swedish Polar Secretariat	Magnus Augner
1G	5.1	Swedish Agricultural University	Goran Bergkvist
1H	-6.1	Swedish Polar Secretariat	Magnus Augner
2C	8.4	Land where there are no restrictions regarding access or soil sampling, and where no endangered species or UK SSSIs (Sites of Special Scientific Interest) were sampled	
2D	8.4	University of Stirling	Philip Wookey
2G	3.1	Land where there are no restrictions regarding access or soil sampling, and where no endangered species or UK SSSIs were sampled	
2H	4.6	Land where there are no restrictions regarding access or soil sampling, and where no endangered species or UK SSSIs were sampled	
3A	10.2	Cranfield University	Jim Harris
3C	10.7	Forestry Commision	Alice Holt Office
3G	9.9	North Wyke, Rothamsted Research	Jennifer Dungait
3H	10.3	RSPB Aylesbeare Common	Toby Taylor
4A	14.3	Tuscia University	Cristina Moscatelli and Sara Marinari
4C	18.5	University of Jaen	Jose Antonio Carreira de la Fuente
4D	15.4	Tuscia University	Cristina Moscatelli and Sara Marinari
4G	16	Forest Sciences Centre of Catalonia (CTFC)	Maria-Teresa Sebastia
4H	16	Forest Sciences Centre of Catalonia (CTFC)	Maria-Teresa Sebastia

### Soil incubation and respiration measurements

The mid- to long-term response of soil microbial respiration to changing temperature was determined using the soil-cooling approach [22], as described in Karhu *et al*. [21]. Briefly, soil replicates from the 20 cores were divided into four treatments: pre-cooling, control, cooled and re-warmed. Control samples were incubated at mean annual temperature (MAT) of their site of origin, plus 3°C for the duration of the whole experiment (174 days). Pre-cooling samples incubated at MAT plus 3°C were destructively sampled after 84 days, when respiration rates had stabilised, and the temperature treatments commenced. Cooled treatment samples were transferred to MAT minus 3°C on day 84 and incubated at that temperature until the end of experiment (day 174). Re-warmed samples were transferred back to MAT plus 3°C after 60 days of cooling, and incubated at MAT plus 3°C until the end of the experiment. Soil respiration data were used to determine whether an enhancing or compensatory community-level response to temperature had altered respiration rates compared to the “no-response” case [21]. The magnitude of enhancing or compensatory responses was calculated as the normalized control respiration rate, at the percentage C loss corresponding to the total percentage C loss in the cooled soils, divided by the normalized cooled respiration rate at the end of the incubation (RR_MT_). Therefore, RR_MT_ describes the microbial community level respiration response to prolonged cooling [21]. Ratios above and below 1 indicate enhancing and compensatory responses, respectively. Ratios close to 1 imply that temperature sensitivity of soil respiration does not change in response to prolonged cooling (i.e. “no-response” soils).

For the purpose of this study, soils were classified into different response groups: (1) soil exhibiting compensatory response (3A), (2) soils showing enhancing responses (1C, 1D, 1G, 1H, 2C, 2H, 4C, 4D), and (3) soils with no-response (1A, 2D, 2G, 3C, 3G, 3H, 4A, 4G, 4H), based on respiration rate data in Karhu *et al*. [21].

### Molecular analysis of microbial community composition and biomass

Sub-samples (250 g of soil sample) for DNA extraction were frozen (-20°C) at the end of the pre-incubation period (pre-cooling, day 84), and at the end of the 174-day incubation period for cooled, control and re-warmed treatments. Frozen samples were shipped in dry ice to the laboratory and then stored at -80°C until DNA extraction. Molecular analysis of DNA extracted from soil microbial communities (multiplex terminal restriction fragment length polymorphism (M-TRFLP), quantitative polymerase chain reaction (qPCR) and amplicon pyrosequencing) did not include tropical soils (hence, *n* = 18). Total DNA from each triplicate was extracted and purified with the UltraClean^®^-htp 96 Well Soil DNA isolation kit according to the manufacturer’s instructions (MoBio Laboratories, USA). The quality of extracted DNA was checked by agarose gel (1%) electrophoresis (100 V, 40 min). Total DNA was quantified in 1 μl DNA samples in 96-well plates with the Quant-iT^™^ PicoGreen^®^ kit (Invitrogen, Canada) according to the manufacturer’s specifications. Fluorescence was quantified using a CytoFluor^®^ 4000 Multi-well plate reader (PerSeptive Biosystems, USA).

Microbial gene abundance, as a proxy of the microbial biomass, was estimated by qPCR for control, cooled and rewarmed soils at the end of the experiment; qPCR assays were performed in triplicate using a CFX96 Touch^™^ Real-Time PCR Detection System (BioRad, Australia). Each qPCR reaction was performed in 20 μl with the QuantiTect^®^ SYBR^®^ Green PCR kit (Qiagen, USA), 250–300 nM of each primer targeting bacterial 16S rRNA gene (1132R, [32]; 1108F, [33]), archaeal 16S rRNA gene (Cren16S957R, [34]; Cren16S771F,[35]) and ITS gene (ITS1F, [36]; ITS2R, [37]) and 10 ng of total DNA previously quantified by Quant-iT^™^ PicoGreen^®^ kit. The amplification conditions were as follows: preheating at 50°C for 2 min, then at 95°C for 15 s followed by 40 cycles at 94°C for 15 s, 60°C for 30 s and 72°C for 30 s. Serially diluted 16S rRNA gene amplicons from *Paracoccus denitrificans* (Bacteria), *Nitrosotalea devanaterra* (Archaea) or ITS gene amplicons from *Suillus variegatus* (Fungi) were used as standards. Melting curves were performed to confirm the purity of the amplified product. Amplification efficiency (*E*) of the primers was within the prescribed values (0.9<*E*<1.1) with linearity (*r*^2^>0.99). Gene abundance is expressed g^-1^ soil dry weight. The sum of gene abundances of Archaea, Bacteria and Fungi was used to estimate total microbial biomass. Fungal:bacterial ratios (F:B) were also calculated based on the qPCR data. M-TRFLP analysis employed universal 16S rRNA gene primers or internal transcribed spacer (ITS) region primers (Applied Biosystems, UK) to target bacterial and fungal communities, respectively, with sample preparation and data analysis as described in Singh *et al*. [38].

Eight soils, representing the full range of respiratory responses and the clearest examples of the different types of response, were selected for more detailed analysis of microbial community composition by pyrosequencing: one soil with compensatory response (3A), three with enhancing responses (1H, 4C, and 4D), and four with no-response (2D, 2G, 3C and 4A). Microbial community composition of each soil was determined in triplicate. Bacteria and archaea were targeted using the universal 16S rRNA gene primers F515 and R805 [39, 40] and fungi using a newly designed ITS primer ITS1F-Kyo1 and ITS2 [36, 41]. Primers were linked to Roche 454 adapters and the multiplex identifiers listed in S1 Table and PCR amplification, purification and quantification were performed as described in SI Material and Methods. The sequence data are available in NCBI’s (National Center for Biotechnology Information) Sequence Read Archive under BioProject PRJNA281794.

### Determination of microbial biomass

Microbial biomass was measured by chloroform fumigation-extraction (CFE) and substrate induced respiration (SIR) at pre-cooling (day 84) and for all treatments (control, cooled, re-warmed) and at the end of the incubation (day 174). Standard fumigation protocols were applied as described in SI Materials and Methods. Microbial biomass was estimated as the difference in DOC concentration between fumigated and control samples (CFE-flush). Given the range of different soil types investigated, no correction factor was applied to these data.

For SIR, soil samples (10 or 30 g for the organic and mineral samples respectively) were amended with either 2 ml of 15 mg glucose C g^-1^ soil C or 2 ml deionized water (control). SIR was calculated as the difference between respiration of glucose-amended and control soils. Measurements were conducted at both the cooled (MAT minus 3°C) and control (MAT plus 3°C) temperatures, with samples being incubated for 24 h or 6 h for soils collected from cooler temperatures (subarctic, Scottish and English soils) or higher temperatures (Mediterranean and tropical soils), respectively. SIR was used to estimate microbial biomass and to calculate mass-specific respiration in non-limiting substrate conditions (R_mass_). SIR (MAT plus 3°C) was divided by CFE biomass, using the approach outlined in Bradford *et al*. [17]. Biomass determined by SIR and CFE was also corrected for C-loss by interpolating between the control sample biomass values measured at pre-cooling and at the end of the experiment. This enabled calculation of control treatment biomass at the same C loss as for the cooled samples at the end of the experiment [21].

### Enzyme activity

Potential enzyme activity was determined for cooled and control treatments at the end of incubation. *β-*glucosidase was chosen as a model extracellular enzyme, because it catalyses the rate-limiting step in cellulose decomposition and is produced by a wide range of microorganisms [42]. The dehydrogenase assay measures intracellular activity and is commonly used to estimate overall microbial activity [43]. Temperature sensitivity was assessed at a temperature range natural to the soil (i.e. reflecting seasonal changes in soil thermal regimes, which vary substantially according to site of origin) by performing assays at four to six temperatures, including MAT plus 3°C and MAT minus 3°C, as used in the long-term incubations.

*β-*glucosidase activity was assayed as described in Alef and Nannipieri [44], except that samples and all reagent and buffer amounts were scaled down to enable the assay to be conducted in Eppendorf tubes. Dehydrogenase activity was assayed using the INT method [45]. A brief description of each protocol is included in SI Materials and Methods. Calibration curves and calculation of results were as described previously [44, 45]. *β-*glucosidase and dehydrogenase activities were efficiently measured in 17 and 13 soils, respectively while both activities were determined in all soils (*n* = 18).

### Data and statistical analyses

The *Q*_10_ values for enzyme activity were calculated from the equation *y* = *ae*^*bT*^, where y = enzyme activity, a = enzyme activity at 0°C, T = temperature in degrees Celsius, *b* = the temperature dependence coefficient, which gives a constant *Q*_10_ = e(10*b). A linearised equation was fitted to ln-transformed enzyme activity data using SPSS linear regression. Baseline enzyme activity (*a*), or its temperature sensitivity (*Q*_10_), were considered to differ statistically significantly between cooled and control treatments if the 95% confidence intervals for the (back-transformed) fitted parameters did not overlap.

Independent samples t-tests were used to detect statistically significant differences in microbial variables (SIR, CFE, or qPCR biomass, R_mass_ or F:B ratios) of control and cooled treatments within each soil at the end of the incubation experiment or between each respiration response group. Similar tests were applied to determine statistically significant differences in the percentage of total OTUs. The significance of differences could not be determined between the compensatory response soil, for which *n* = 1 as previously demonstrated by Karhu *et al*. [21], and the no-response or enhancing response soils. Linear regression analysis was used to relate the magnitude of the respiration responses (RR_MT_) in different soils to changes in these microbial parameters, soil properties and other parameters describing microbial community composition across all data. Analyses were conducted using SPSS Statistics 22 (IBM, USA).

Statistical analysis of the M-TRFLP samples was based on the complete sample profiles as expressed by the pattern of M-TRFLP peaks and the relative abundance (percentage) of individual peaks in each profile [38]. To determine whether information on the initial microbial community composition could help to predict the respiratory response, Canonical Variates Analysis (CVA) was performed on M-TRFLP data (precooling treatment samples) using GenStat 14^th^ edition (VSN International Ltd, UK) and coordinates were obtained (S1 Fig). Each CVA axis shows the best separation between groups defined by their respiratory response and using microbial community structure data obtained at the end of the incubation.

Pyrosequencing data analysis was performed using the ‘Quantitative Insights Into Microbial Ecology’ (QIIME v1.6.0) software package [46]. Strict quality filtering steps were performed before affiliation to phylogenetic clusters and operational taxonomic units (OTU; SI Materials and Methods).

## Results

### Differences in initial microbial community composition

**Fig 1:**
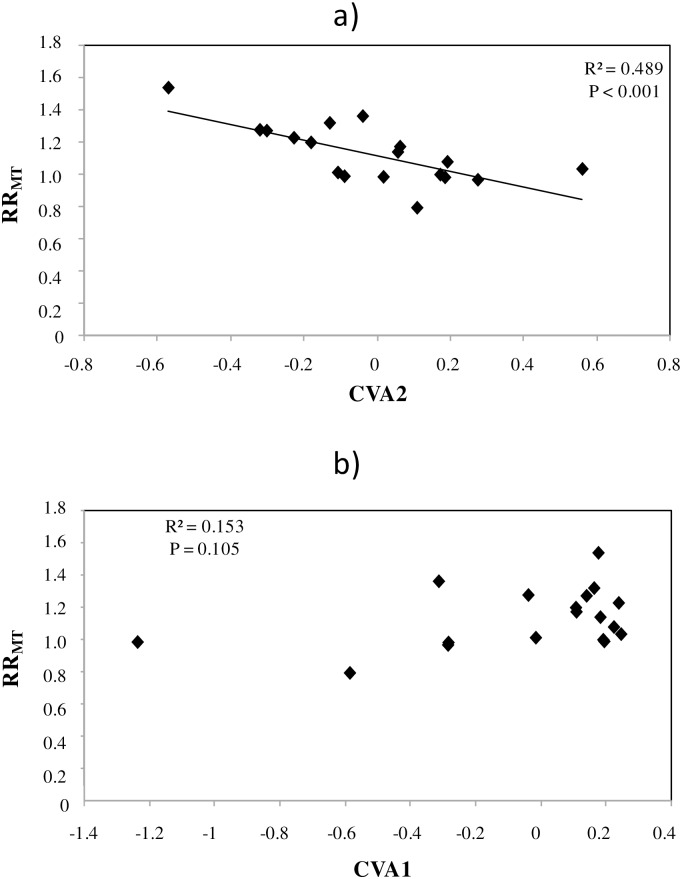
Linear regressions between a) RR_MT_ and CVA1 or b) CVA2. Black diamonds represent each soil (n = 18).

CVA did not separate clearly the initial microbial community structures of soils associated with different respiration responses (S1 Fig), but there was a significant correlation between the initial microbial community structure (the second CVA axis) and RR_MT_ (Fig 1a). This explained 49% of the variability observed on this axis, which discriminates soils showing enhancing response from no-response. In contrast, CVA1 and RR_MT_ (Fig 1b) were not significantly correlated. Although C:N ratio in soils had previously been found to be related to the respiration response [21], microbial community (CVA1 or CVA2) and C:N ratio were not correlated when all soils were tested (S2a and S2b Fig).

### Changes in microbial community composition (based on pyrosequencing) in response to cooling

**Fig 2:**
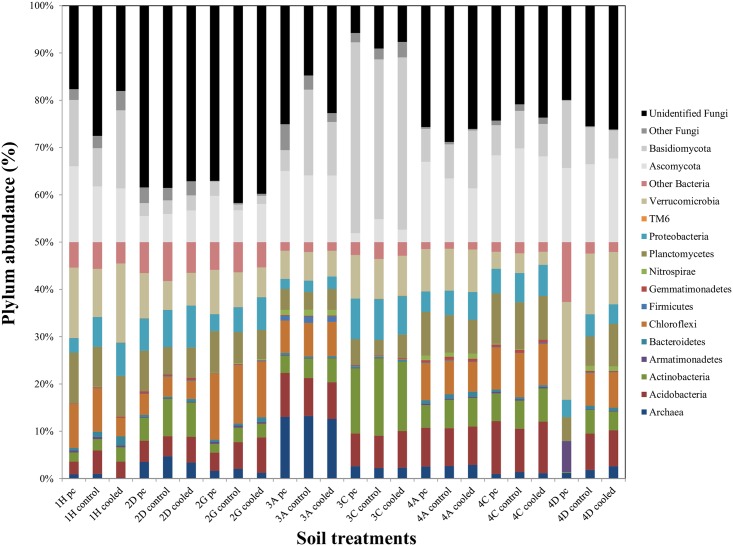
Community composition based on pyrosequencing. The relative abundance of each microbial phylum (Archaea, n = 1; Bacteria, n = 13; Fungi, n = 4) is indicated for precooling (pc), control and cooled treatments.

Generally, OTUs associated with *Actinobacteria*, *Acidobacteria*, *Chloroflexi*, *Planctomycetes* and *Verrucomicrobia* phyla had the highest relative abundance (Fig 2) and *Actinobacteria* comprised mainly (approximately 80%) *Actinomycetes*. The relative abundance of *Actinobacteria* OTUs was significantly higher in 3C (no-response) than in other soils, comprising almost 22% of the bacterial community (control). For fungi, *Ascomycota* and *Basidiomycota* OTUs were generally dominant or comprised at least 50% of the fungal community except in 2D and 2G (no-response soils), which were dominated by unidentified fungi. Moreover, *Basidiomycota* OTUs were negatively correlated (*P*<0.01) with the respiration response (S2 Table). The most dominant *Basidiomycota* species in 3A belonged to the species *Clitopilus*, a saprophytic genus, (12.3 ± 6.1% of the entire fungal community), while *Basidiomycota* dominating fungi in 3C and 1H were ectomycorrhizal fungi. In contrast, *Ascomycota* were dominant in most Mediterranean soils: 4A (no-response) and 4C and 4D (enhancing responses). Archaeal OTUs were a minor component, representing, on average, 4% of all prokaryotic sequences.

**Fig 3:**
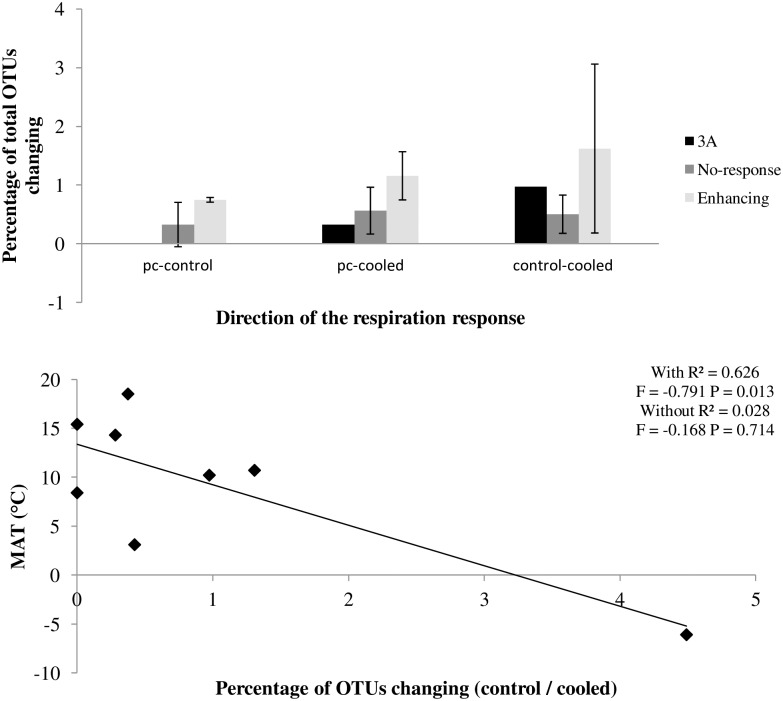
Number of OTUs changing (mean ± S.E) in response to time of incubation (comparing precooling vs. control) or cooling (comparing cooled vs. control soils) or both (comparing precooling vs. cooled). a) in different respiration response groups, b) linear regression between the number of changing OTUs and site MAT with or without 1H. Grey bars represent each group with standard error bars. Error bars for compensatory group were not added (n = 1). Black diamonds represent each soil (n = 8).

Potential rates and drivers of change in microbial community composition were explored by comparisons, at the end of the experiment, of: 1) pre-cooling and control soils (C and nutrients availability effect); 2) pre-cooling and cooled soils (temperature effect) and 3) control and cooled soils. Generally, the relative abundances of OTUs did not change greatly after cooling or after incubation in all soils tested and were restricted to <2% of total OTUs (Fig 3a and S3 Fig, S3 Table). No significant differences were observed when comparing the percentage of OTUs changing between soils with enhancing response and soils with no-response. The greatest number of OTUs changing was observed in 1H (S3 Table), an arctic soil exhibiting a strong enhancing response, contrasting with some Mediterranean soils with enhancing responses (4C, 4D). The average percentage of OTUs changing (control compared to cooled treatment) was also inversely related to site MAT (Fig 3b), although this correlation was no longer significant after removal of one arctic soil (1H). Cooling had a limited effect on F:B ratio, determined using qPCR data corrected for C loss (S4 Fig), increasing only in a limited number of soils, and showing no correlation with RR_MT_ or C:N ratio.

### The effect of temperature on microbial biomass and R_mass_

**Fig 4:**
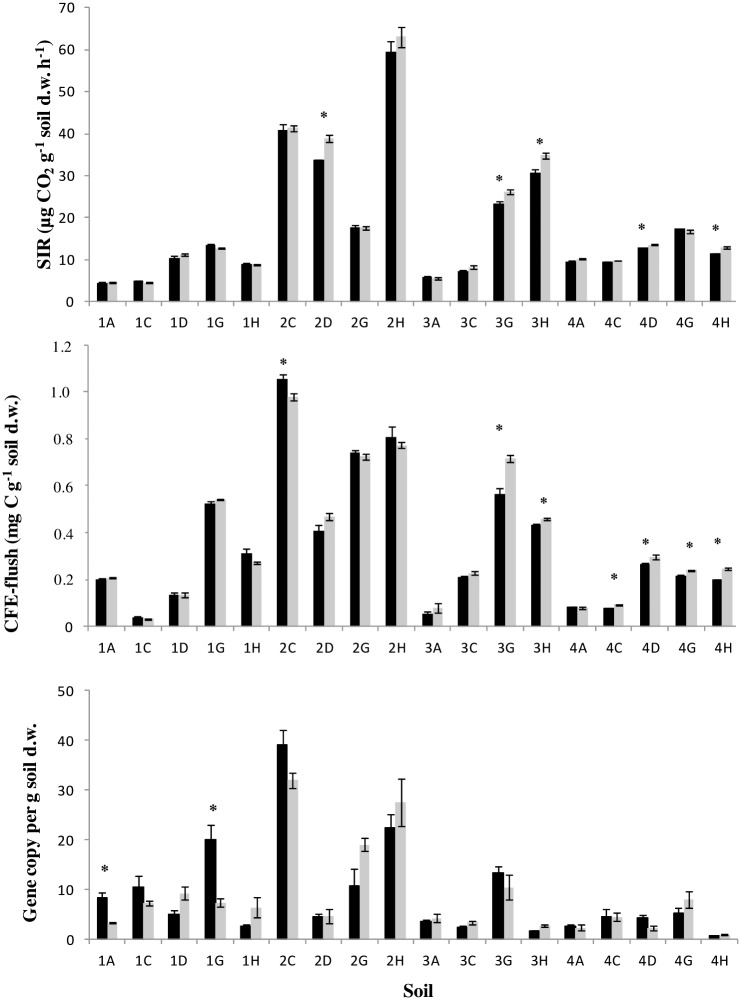
Microbial biomass estimated using a) SIR, b) CFE and c) qPCR methods and corrected for carbon loss in the control treatment (interpolated between pre-cooling and control treatment end of experiment biomasses, so that the MAT plus 3°C soils were compared to the cooled (MAT minus 3°C) soils at a similar C loss, corresponding to the maximum C loss of cooled samples at the end of the experiment. Each soil is identified by geographic/climatic area including 1 (subarctic), 2 (Scotland), 3 (England), 4 (Mediterranean) and 5 (tropical), and ecosystem type: A (arable), C (coniferous evergreen forest), D (deciduous broadleaf forest), G (grassland), H (ericaceous heath) and E (evergreen broadleaf forest) (21). Mean ± S.E. is presented (n = 3). Black bars (control treatment), grey bars (cooled treatment). Statistically significant differences (*P*<0.05) are marked with an asterisk.

Overall, CFE microbial biomass was lower in the control soils at the end of the incubation, but when corrected for differences in total C loss, cooled and control soils did not differ significantly. This indicates that these differences were due to different labile C availability (reduced total C loss in the cooled samples), and not to a direct effect of temperature *per se*. C loss corrected CFE-biomass differed significantly (*P*<0.05) between some individual soils (Fig 4) and, in general, changes in biomass could not explain the observed respiration responses. Even when small differences in biomass of cooled and control treatments were found, differences were not in a consistent direction.

**Fig 5:**
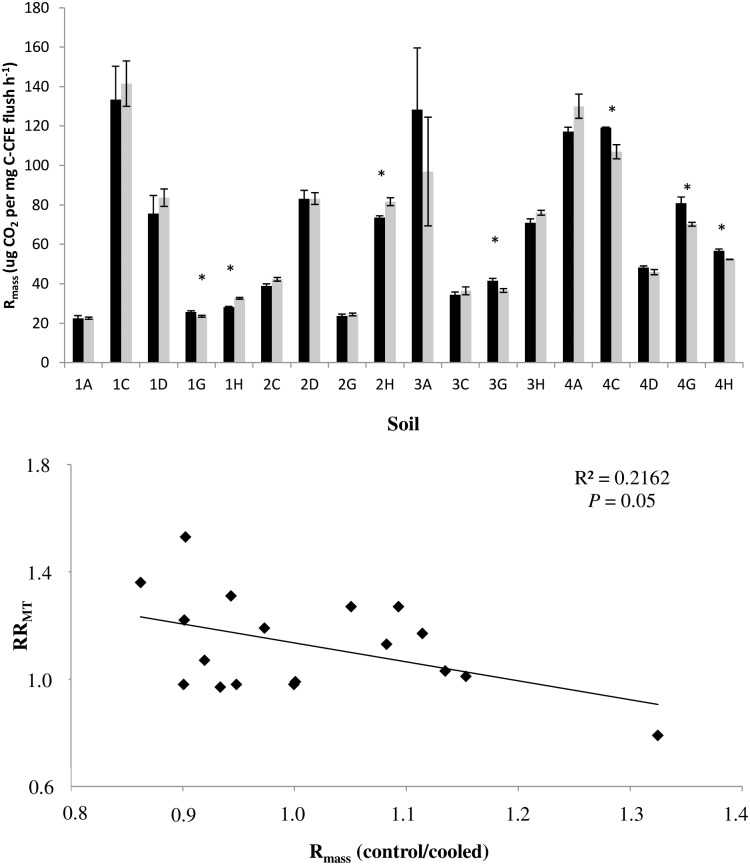
a) **R**_**mass**_ at the end of the experiment for control (black bars) and cooled treatments (grey bars). Statistically significant differences (*P*<0.05) are marked with an asterisk. b) **R**_**mass**_ **ratio (control/cooled) regression with RR**_**MT**_. Black diamonds represent each soil (n = 20). Code for soil origin provided in the legend to Fig 1.

In contrast to previous studies, cooling did not increase substrate-unlimited, biomass-specific respiration (R_mass_; SIR/CFE at the end of the experiment) in the soil with a compensatory response (3A) (Fig 5a). In addition, there were a few statistically significant differences in R_mass_ of cooled versus control treatments in soils exhibiting no-response or soils with enhancing responses, and, overall, there was a weak negative relationship between R_mass_ and RR_MT_ (Fig 5b). In addition, as highlighted by Karhu *et al*. [21], it should be emphasised that when RR_MT_ (i.e. not substrate-induced respiration) was divided by SIR, CFE or qPCR biomass, the overall patterns did not change and enhancing responses remained much more frequent than compensatory responses.

### Enzyme activities

*β-*glucosidase and dehydrogenase activities in control and cooled treatments were not statistically different (S5 and S6 Figs, S4 and S5 Tables) and enzyme activity per CFE microbial biomass (parameter *a*, activity at 0°C) was not related to site MAT. The *Q*_10_ values for *β-*glucosidase and dehydrogenase activities varied between 1.5 and 2.2 (S4 Table), and 1.3 and 3.1 (S5 Table), respectively, and did not differ between cooled and control treatments within each soil. The only exception was a higher *Q*_10_ value for dehydrogenase activity in cooled treatment of soil 2G, a no-response soil (S5 Table). There was no correlation between site MAT and *Q*_10_ values across all data.

## Discussion

### The role of the initial microbial community composition in explaining enhancing, compensatory or no-response

Our results suggest that the initial composition of the soil microbial community influenced the respiration responses observed, although unmeasured variables (e.g. soil or environmental factors) could have affected both microbial community composition and respiration response [47, 48], and interrelationships do not necessarily imply causality. However, the strong correlation between RR_MT_ and the second CVA axis values indicates that some component of the microbial community affects the community’s capacity to react to prolonged temperature changes. Finally, a correlation between microbial community composition and soil C:N ratio was not detected, contrasting with Karhu *et al*. [21], who reported C:N ratio to be the only soil or site variable correlated with the RR_MT_ responses across all data. Karhu *et al*. [21] confirmed that the average RR_MT_ values were greatest for soils with a MAT below 7°C or above 14°C. Therefore, microbial community composition and some environmental factors interacted directly or indirectly to influence respiration rates, as confirmed by Matulich and Martiny [49].

The community in soil with compensatory response (3A) did not seem to differ from other soils (along the first CVA axis) at the initial stage or after cooling, but statistical analysis could not be performed, as this was the only soil exhibiting a compensatory response. However, this soil was dominated by a limited number of *Basidiomycota*, mostly saprophytic fungi, while the other soils were dominated by ectomycorrhizal fungi. Fungi are important for decomposition of recalcitrant C and Cline and Zak [50] confirmed the importance of the initial fungal colonizers in structuring microbial communities and subsequently influencing organic matter decomposition rate. In single-species cultures, respiration of different *Basidiomycota* species has been shown to acclimate to temperature [24] and there have been suggestions that fungal dominance can enhance C sequestration [51]. Both fungal phyla were identified using a DNA-based method that does not give information on microbial activity, and it is not possible to confirm the importance of saprophytic *Basidiomycota* for controlling microbial respiration based on this method, but the result suggests that further work is required to link respiratory responses to temperature to particular functional groups of microbes.

### Shifts in microbial community composition with temperature change

Generally, there were few changes in community composition between pre-cooling and the end of the experiment in the control treatments. This suggests that the microbial communities were already well adapted to the MAT + 3°C temperature during the pre-incubation period and that the approach adopted minimised changes in labile C availability/SOM quality after the pre-incubation period. This was also supported by the stabilisation of respiration rate during the 84-day pre-incubation period [21]. Also, there were no significant differences between soil microbial communities in cooled and pre-cooled treatments. Moreover, changes in fungi:bacteria ratio associated with a specific respiration response were inconsistent, suggesting that control of the respiration response may result from higher activity of some microbial degraders. Therefore, it appears that the initial microbial community remained stable even after temperature change or a gradual loss of soil C. This result may indicate that microbial community composition under temperature change is not the sole determinant of the diverse microbial respiration responses detected, and that the responses observed may be determined by other factors, such as interactions between species, soil properties and disturbance history [52]. Moreover, our molecular biology-based results were obtained after analyzing DNA only, and therefore give no information on active populations.

Some support was obtained for our prediction of greater changes in microbial community composition at lower temperatures, with site 1H showing a particularly large change, mostly in fungal populations that may involve cold-adapted fungi [53]. As suggested by Rinnan *et al*. [54], bacterial populations from colder regions were less impacted by temperature. This suggests that temperature represented a much stronger selection pressure at low temperatures, with this potentially helping to explain the greater respiratory responses observed in high-latitude soils [21]. However, further work is required to explore how the developing cold tolerance affects respiratory responses to warming in different microbial groups.

### Biomass and biomass specific respiration rates and enzyme activity

There were no changes in microbial biomass that could explain the overall patterns of respiration responses observed, and the pattern of responses persisted when expressed per unit biomass [21]. This emphasises that changes in total microbial biomass were not the key factor controlling the sign or magnitude of the microbial community response. As indicated above, analysis of only DNA provides no information on active members of the community and further work is required to determine if changes in active microbial biomass could be involved [55]. It has also been argued that thermal adaptation should be investigated in conditions where substrate availability is non-limiting (R_mass_, [17]), and substrate induced-respiration has been expressed per unit microbial biomass to test this. However, no changes were observed in R_mass_ that could explain the patterns observed in respiration responses. Our approach largely accounts for changes in substrate availability, suggesting that changes in R_mass_ observed in previous studies that were attributed to thermal adaptation [17, 56] may have been related more to changes in labile C availability following long-term warming [17], or in response to sugar addition [56].

Microbial models have predicted that “thermal acclimation” (i.e. compensatory response) should lead to decreased enzyme pool sizes with warming [57], but no differences were found in potential enzyme activity between cooled and control soils. This could indicate that C is allocated to enzyme production prior to growth, as suggested by Steinweg *et al*. [31], and our results suggest that enzyme activities were not directly proportional to biomass, in agreement with Allison [58] and Moorhead *et al*. [59]. Moreover, no change was observed in the temperature sensitivity of enzyme activity with cooling and *Q*_10_ values varied within similar ranges to those reported in earlier studies [30, 60, 61]. Measuring potential enzyme activity does not provide the information that is required to evaluate fully the effects of temperature on enzyme production and stability, and detailed studies of enzyme kinetics are required to advance understanding of long-term temperature responses.

To summarise, our results have advanced our understanding of potential mechanisms underlying microbial respiration responses to changes in temperature. Initial microbial community composition seems to play an important role in determining the respiration response, although the possibility of unmeasured factors affecting both community composition and respiratory response cannot be ruled out. However, there was no support for the hypothesis of greater change in community composition with cooling in all soils exhibiting enhancing responses than in soils with no-responses. In contrast to the apparent importance of the initial microbial community composition, changes in microbial biomass, substrate-unlimited mass-specific respiration or potential enzyme activities did not appear to underpin the respiratory responses. Further study is required to identify the role of specific groups in promoting enhancing versus compensatory responses, such as a potential role of saprophytic *Basidiomycota* indicated here. If this could be achieved then it may be possible to identify ecosystems in which the soil C stocks may be vulnerable or resistant to future climate warming.

## Supporting Information

S1 Fig Canonical Variate Analysis (CVA) based on M-TRFLP results. Black open square: compensatory response (3A), black open triangles: enhancing responses, black crosses: soils with no-response.
(PDF)

S2 Fig Linear regressions between soil C:N ratio and CVA2 (n = 18). Grey diamonds represent each soil (n = 18).
(PDF)

S3 Fig Number of total OTUs in precooling (pc), control and cooled treatments. (PDF)

S4 Fig Fungal to bacterial ratio (F:B) for control and cooled treatments grouped by respiration responses. Dark grey = control treatment, Grey = cooled treatment. C: compensatory; E: enhancing; N: no-response. Mean ± S.E. is presented. * indicate significant differences (*P* < 0.05) following t-tests.
(PDF)

S5 Fig Beta-glucosidase activity per CFE biomass (mg C, expressed as CFE-flush with no correction factor used). Black diamonds = control treatment, open diamonds = cooled treatment. Mean ± S.E. is presented (n = 3). PNP: *p*-nitrophenyl-beta-D-glucopyranoside.
(PDF)

S6 Fig Dehydrogenase activity per CFE biomass (mg C, expressed as CFE-flush with no correction factor used). Black diamonds = control treatment, open diamonds = cooled treatment. Mean ± S.E. is presented (n = 3). INF: iodonitrotetrazolium formazan.
(PDF)

S1 File SI Materials and Methods. (DOCX)

S1 Table List of 454 sequencing (Roche) adaptors used. (DOCX)

S2 Table Spearman correlation results. * Spearman correlation results differ at °*P*<0.1, or *P<0.05. ^a^ The number of soils tested is n = 8. ^1^ Values obtained from bulk soils initially collected. ^2^ Values based on the ratio between control and cooled treatments.
(DOCX)

S3 Table Significantly different OTUs (control / cooled treatment) determined by pyrosequencing. D.L: Below the detection limit; Underlined phyla represent fungal phyla; ^a^MBGA: Marine Benthic Group A of Archaea. * indicates that higher OTU abundance was significantly different between treatments, confirmed by Bonferroni and FDR tests (*P*<0.05). pc: precooling.
(DOCX)

S4 Table Temperature sensitivity of *β-*glucosidase activity expressed per chloroform fumigation extraction biomass (μg PNP mg^-1^ CFE-flush h^-1^). Temperature range used for determining *Q*_10_ of enzyme activity was 1 to 29°C for soils 1A to 2H, 1 to 19°C for 3G soil, 1 to 13°C for 3H soil, and 1 to 32°C for soils 4A to 4H. These different temperature ranges were selected to reflect seasonal temperature changes in soils depending of the site of origin.
(DOCX)

S5 Table Temperature sensitivity of dehydrogenase activity expressed per chloroform fumigation extraction biomass (μg INF mg^-1^ CFE-flush h^-1^). *Q_10_ values differ at the 0.05 level (95% confidence intervals do not overlap).
(DOCX)
