# Supplementary figures and images for: The Role of Microbial Community Composition in Controlling Soil Respiration Responses to Temperature
Source: PLoS One. 2016 Oct 31;11(10):e0165448. doi: 10.1371/journal.pone.0165448 (PMC5087920; doi:10.1371/journal.pone.0165448)

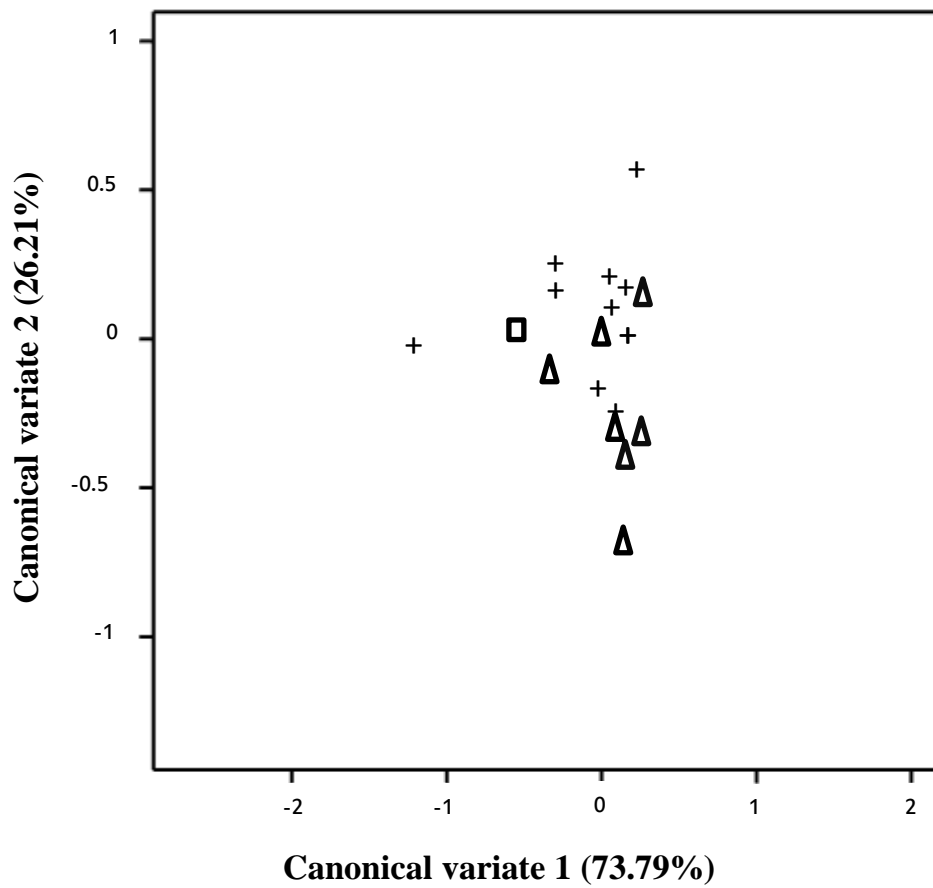

Supplement: S1 Fig — Black open square: compensatory response (3A), black open triangles: enhancing responses, black crosses: soils with no-response. (PDF) [file pone.0165448.s001.pdf]

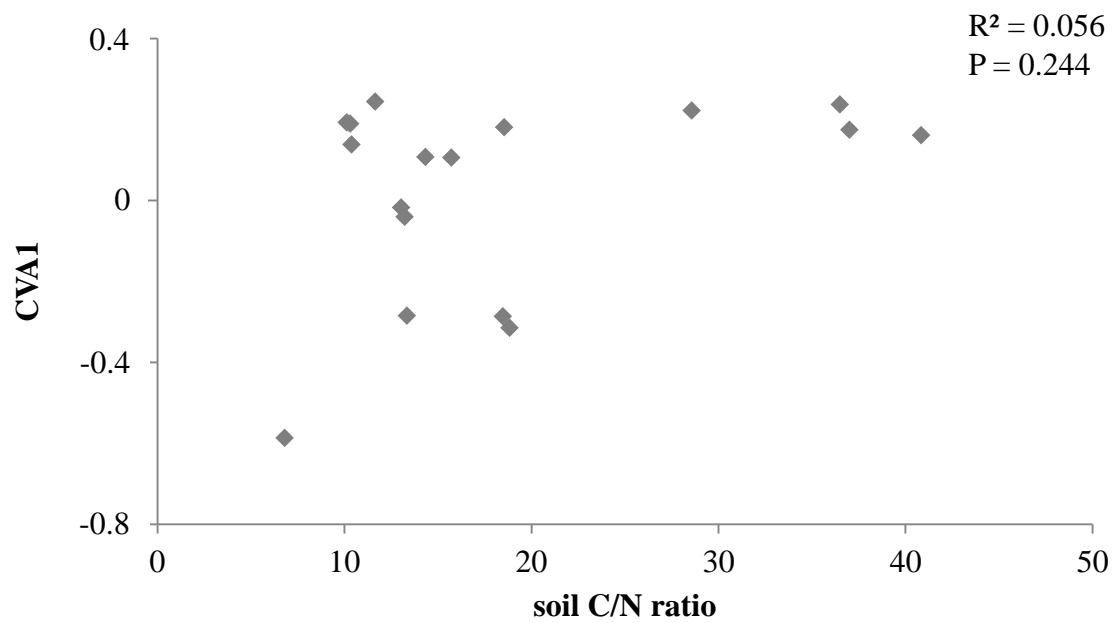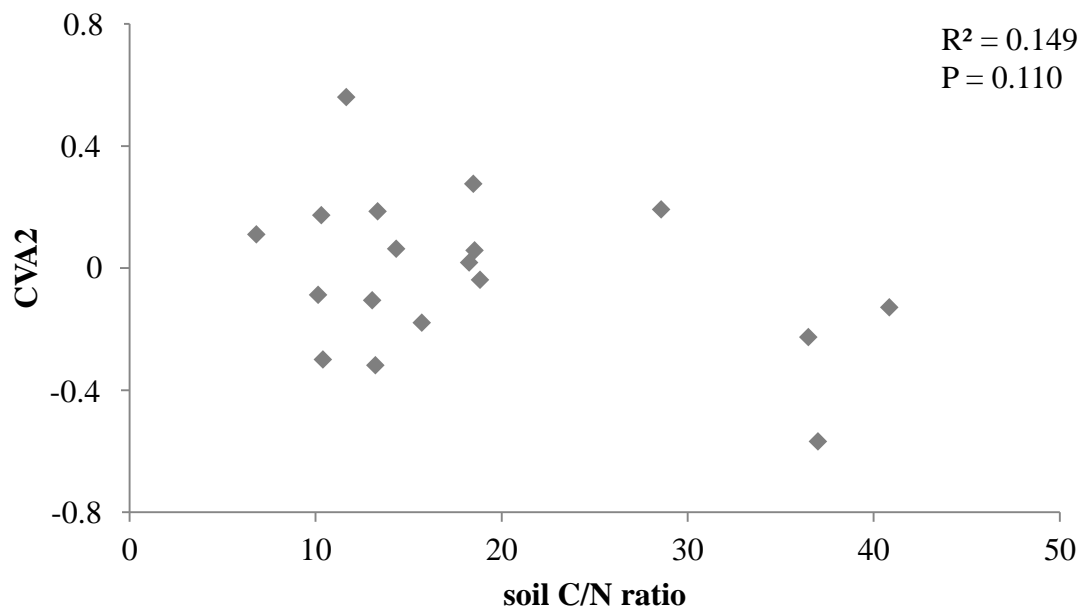

Supplement: S2 Fig — Grey diamonds represent each soil (n = 18). (PDF) [file pone.0165448.s002.pdf]

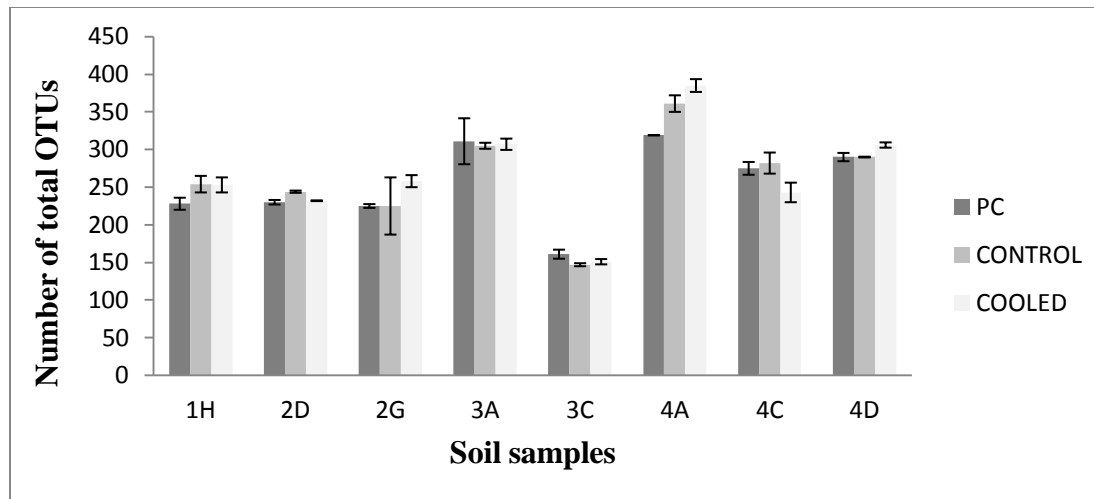

Supplement: S3 Fig — (PDF) [file pone.0165448.s003.pdf]

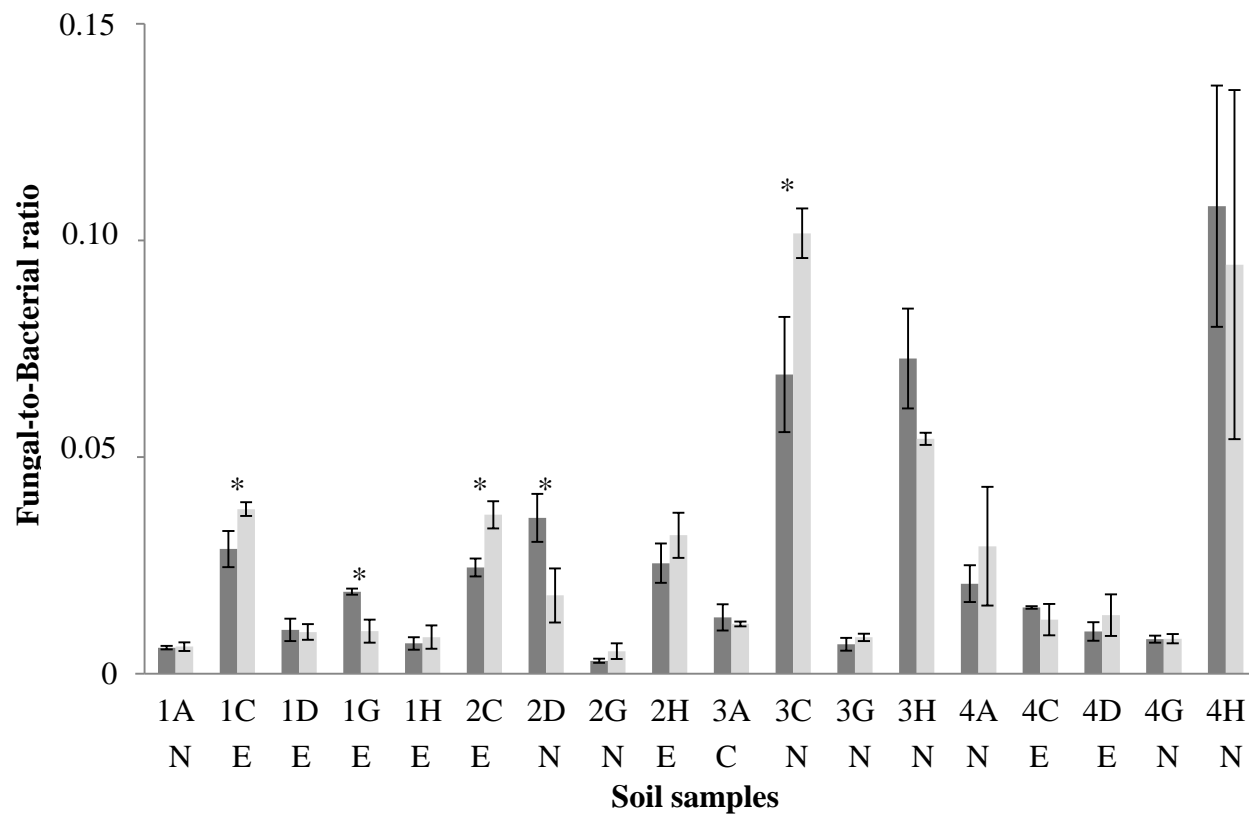

Supplement: S4 Fig — Dark grey = control treatment, Grey = cooled treatment. C: compensatory; E: enhancing; N: no-response. Mean ± S.E. is presented. * indicate significant differences (P < 0.05) following t-tests. (PDF) [file pone.0165448.s004.pdf]

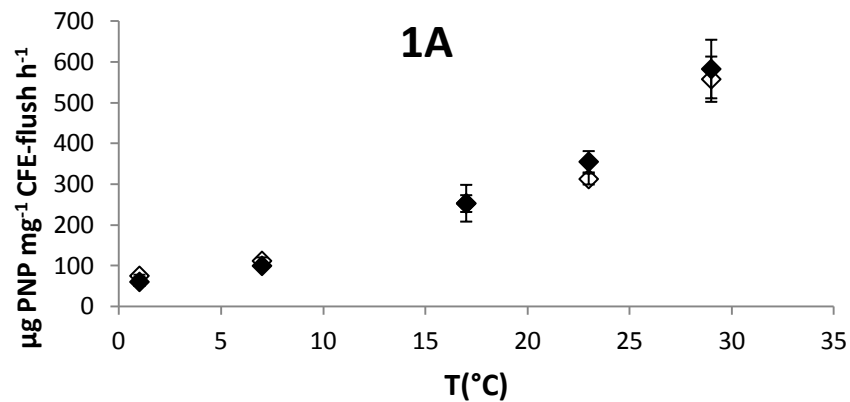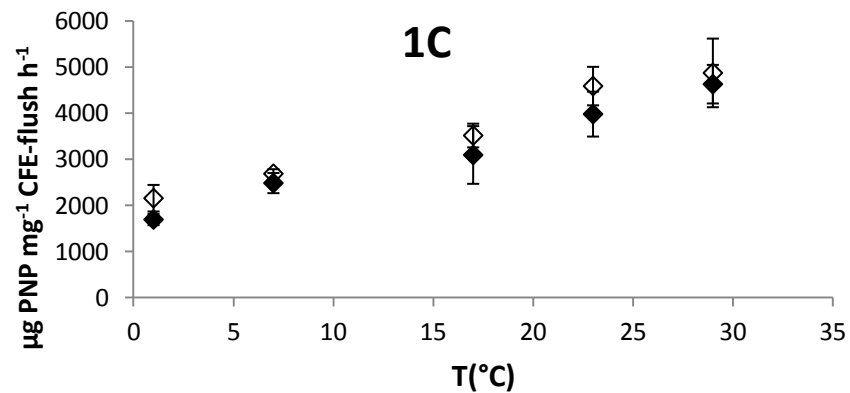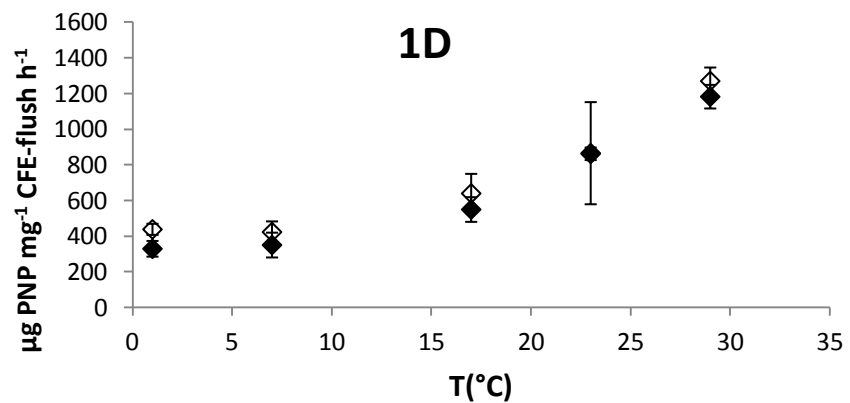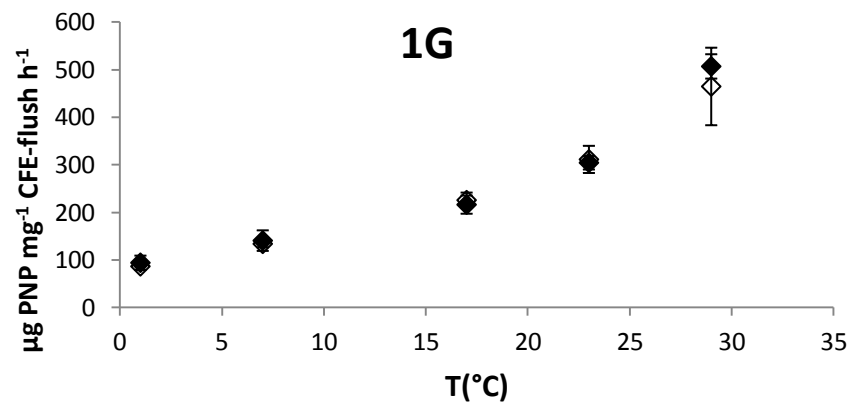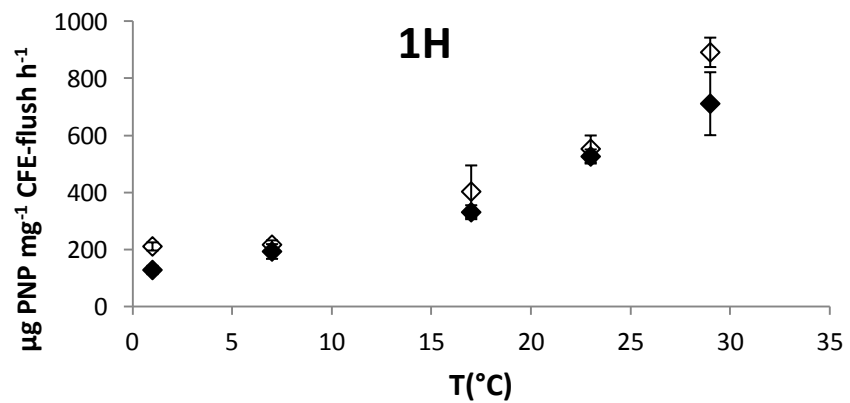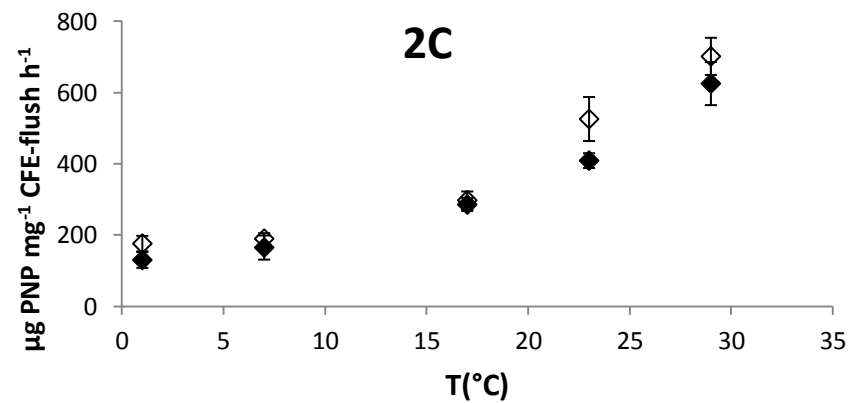

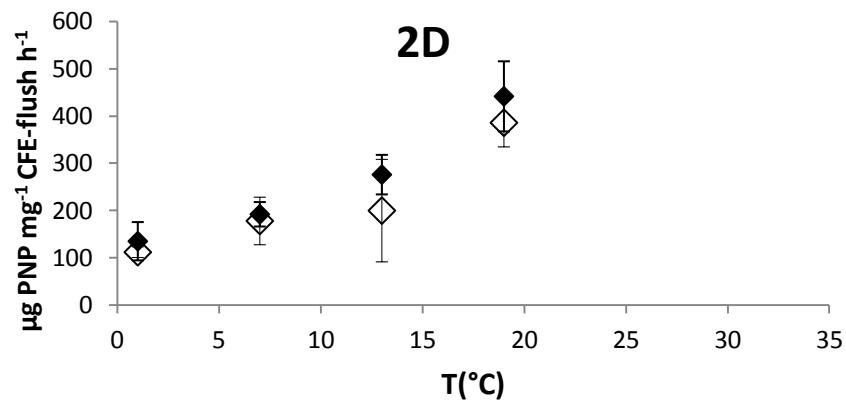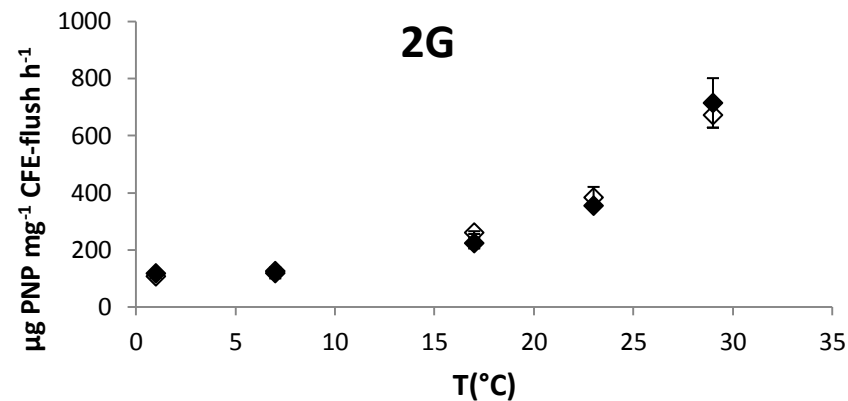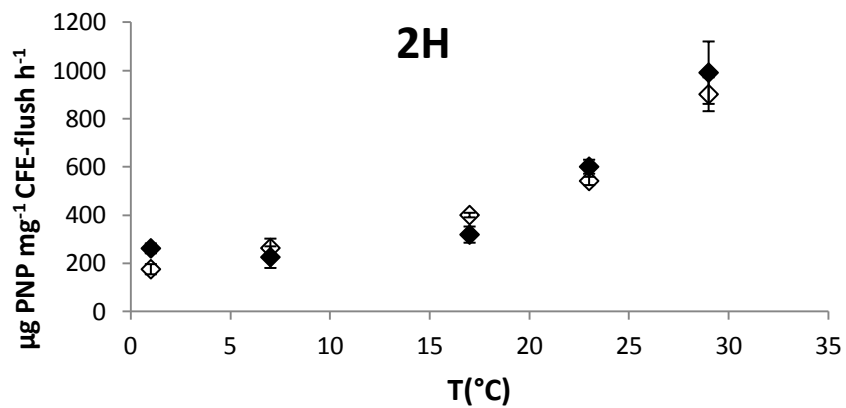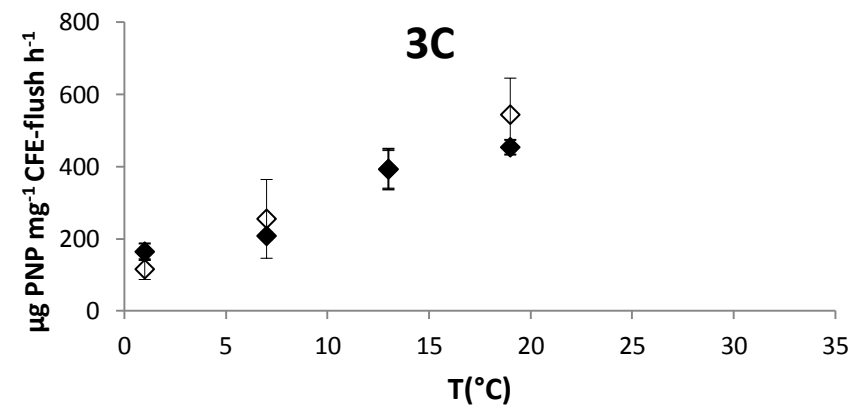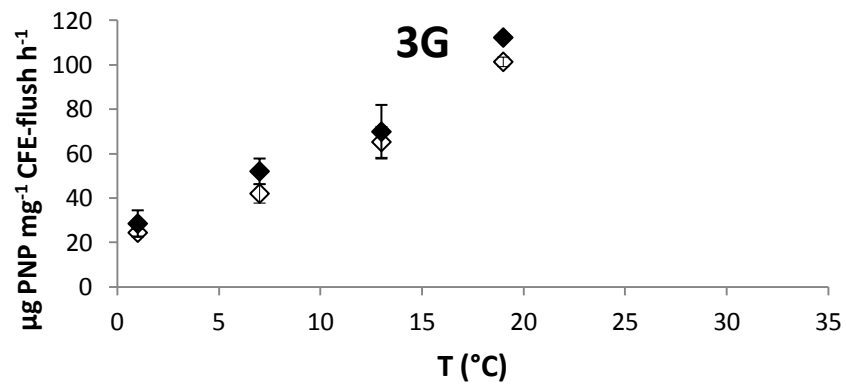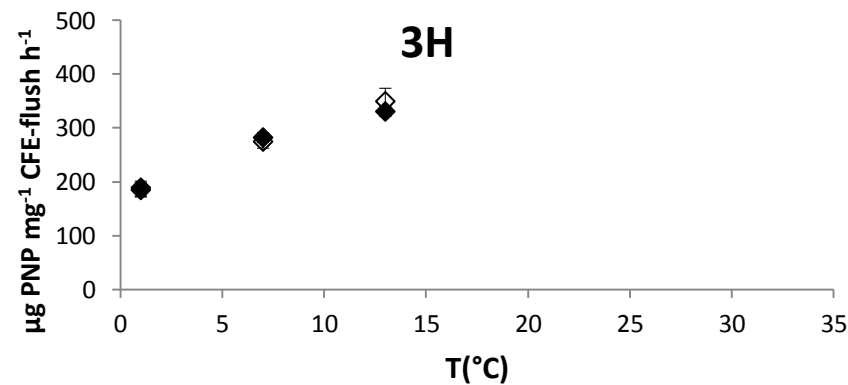

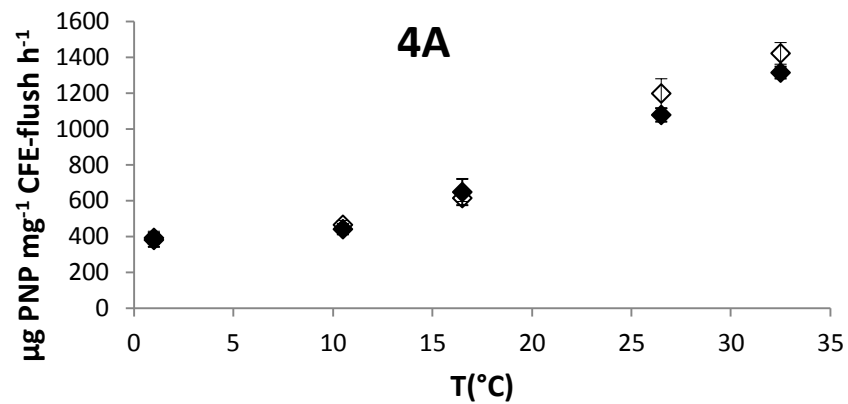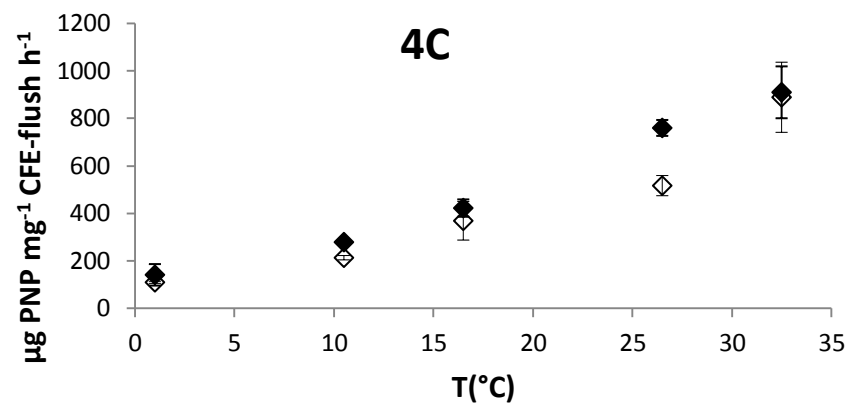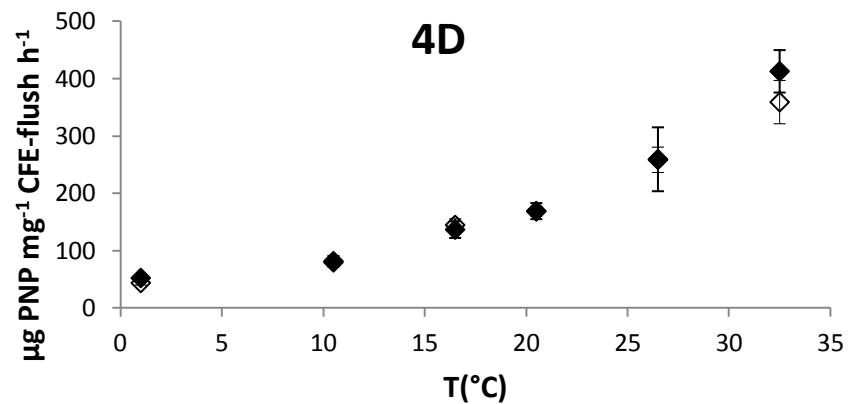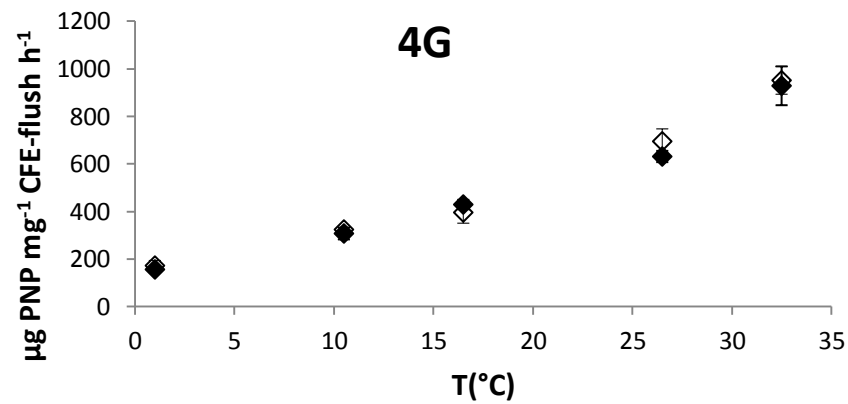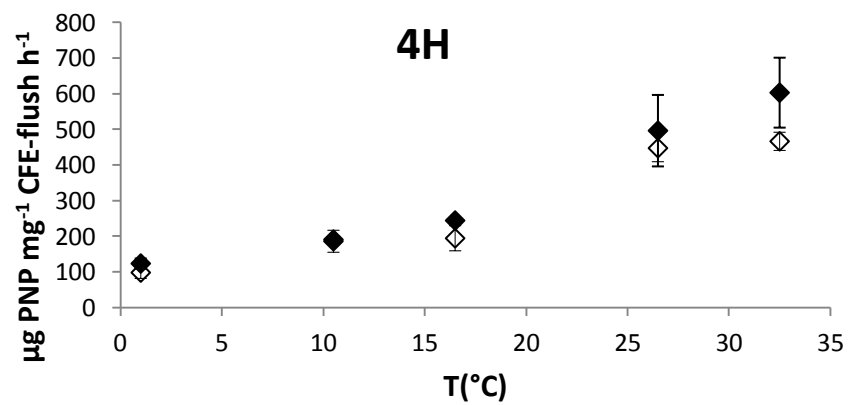

Supplement: S5 Fig — Black diamonds = control treatment, open diamonds = cooled treatment. Mean ± S.E. is presented (n = 3). PNP: p-nitrophenyl-beta-D-glucopyranoside. (PDF) [file pone.0165448.s005.pdf]

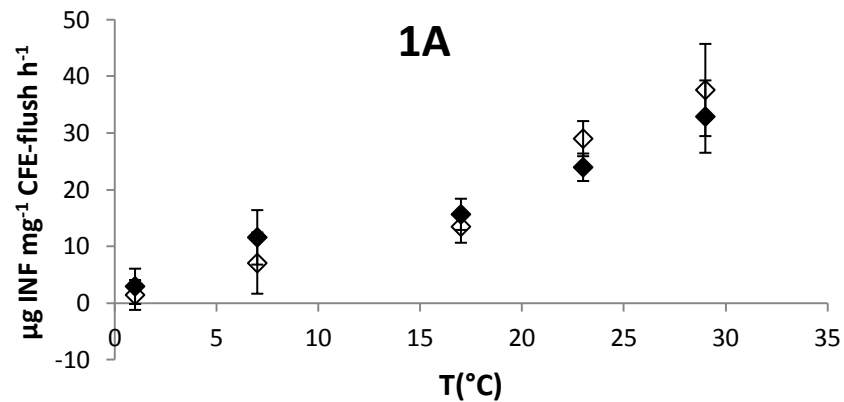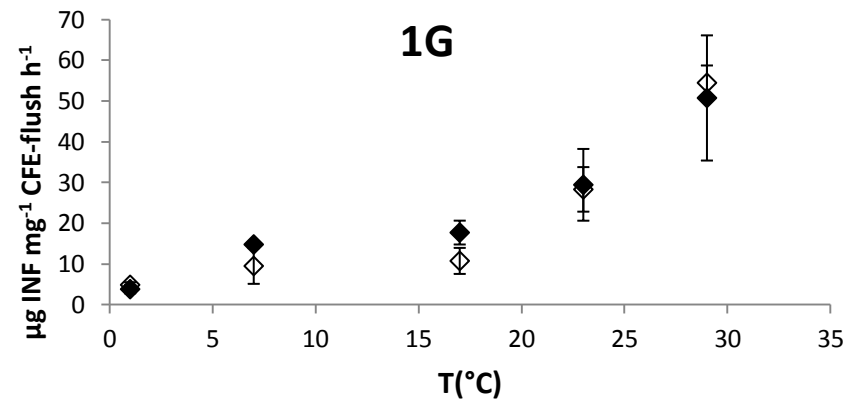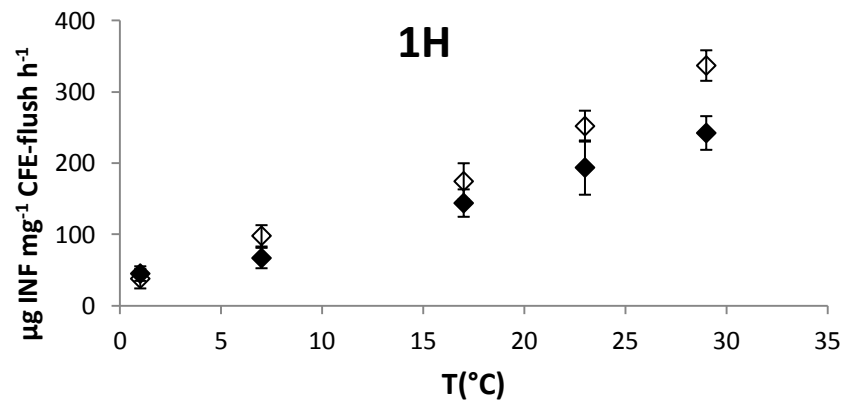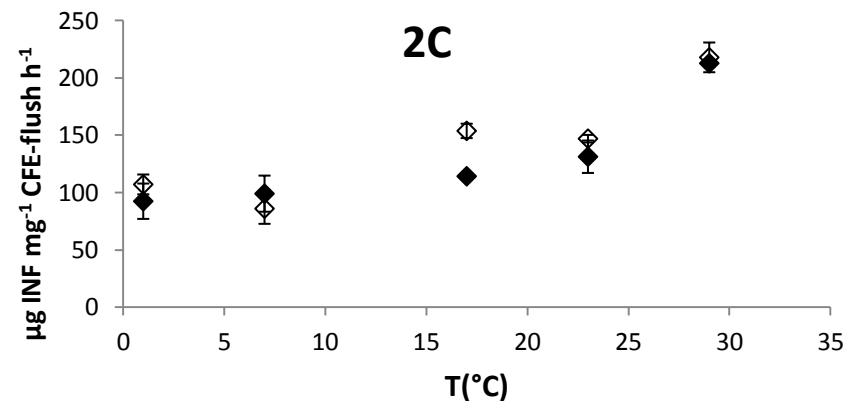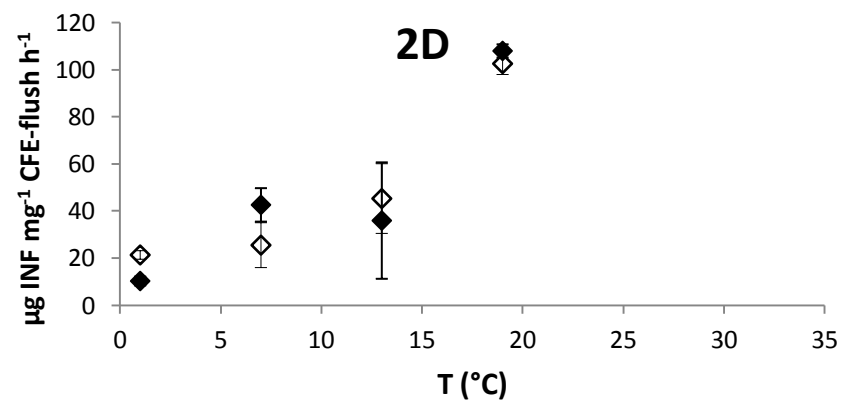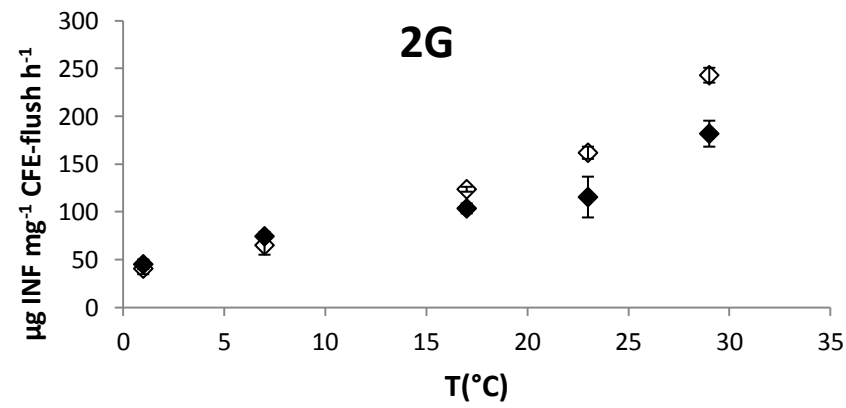

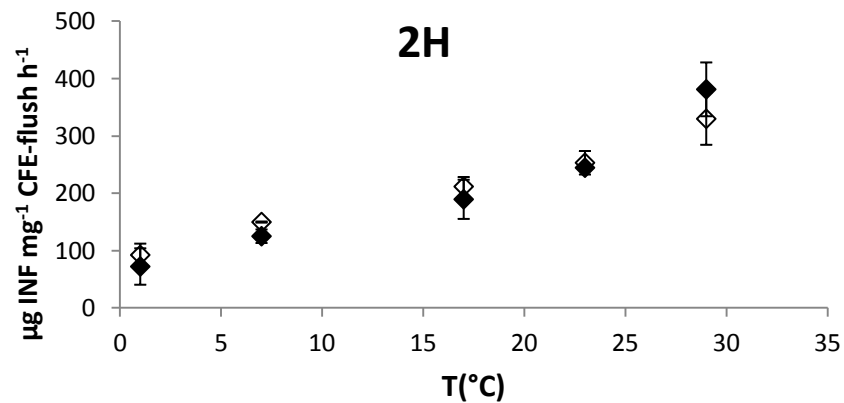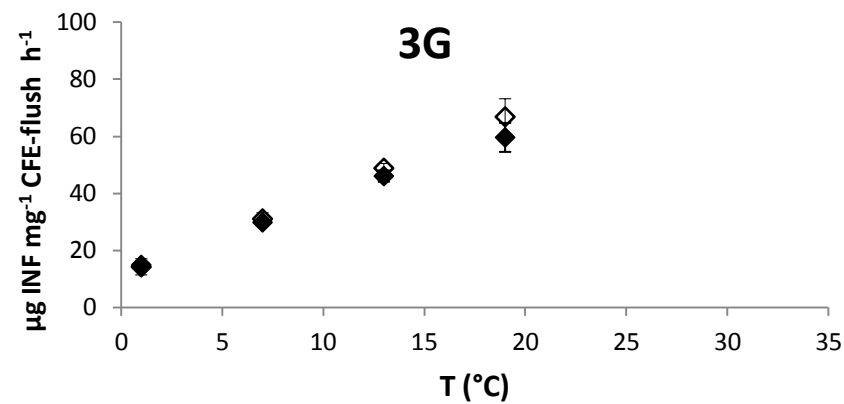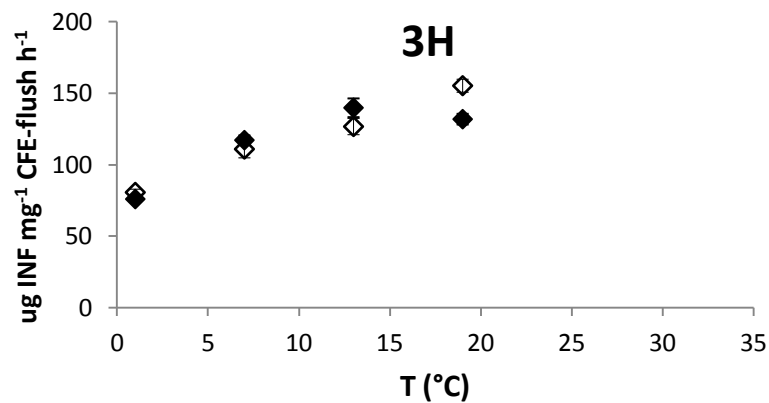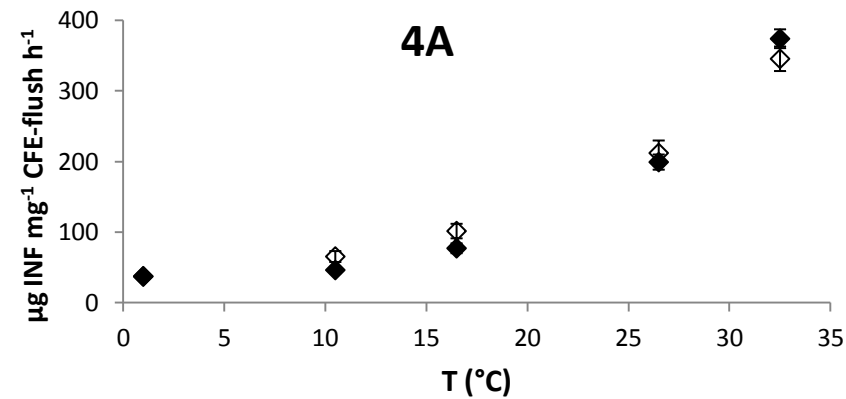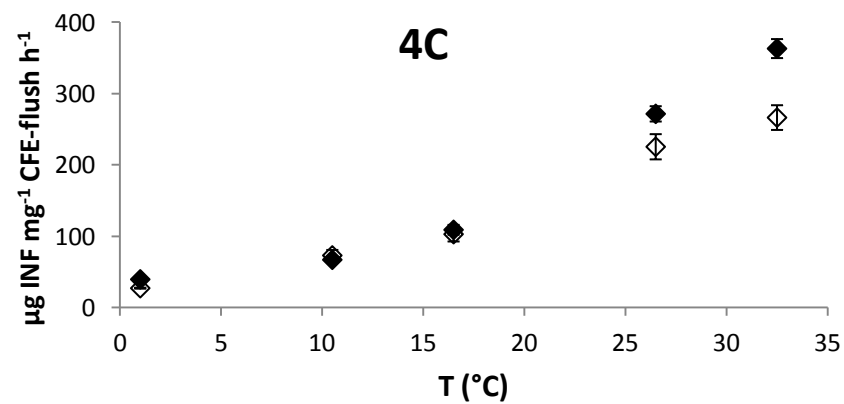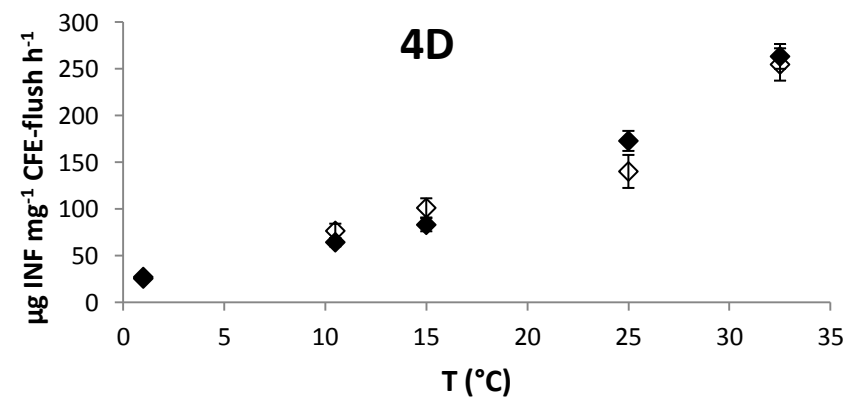

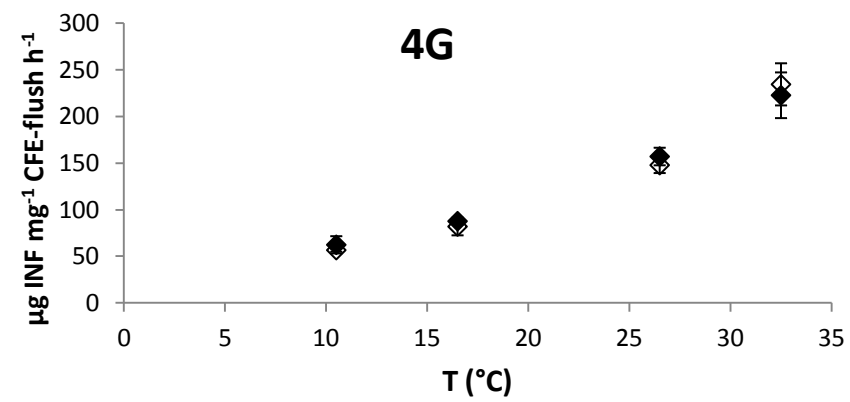

Supplement: S6 Fig — Black diamonds = control treatment, open diamonds = cooled treatment. Mean ± S.E. is presented (n = 3). INF: iodonitrotetrazolium formazan. (PDF) [file pone.0165448.s006.pdf]
