# Supplementary material for: The Role of Microbial Community Composition in Controlling Soil Respiration Responses to Temperature
Source: PLoS One. 2016 Oct 31;11(10):e0165448. doi: 10.1371/journal.pone.0165448 (PMC5087920; doi:10.1371/journal.pone.0165448)
Supplement: S1 File — (DOCX) [file pone.0165448.s007.docx]

**Supplementary information**

**Material and Methods**

**Sample preparation for M-TRFLP analysis**

Polymerase chain reaction (PCR) amplification was performed with a DNA Engine Dyad Peltier Thermal Cycler (Bio-Rad, Australia) and M-TRFLP profiles were obtained as previously described [1]. Fragment size analysis was carried out with an ABI PRISM 3130xl genetic analyzer (Applied Biosystems, UK). Bacterial and fungal profiles were produced using GeneMapper software (version 4.0; Applied Biosystems, UK) and fragments quantified using the advanced mode and second-order algorithm. Fragment analysis was performed using settings described in Singh *et al.* [1]. Briefly, the relative abundance of a terminal restriction fragment (TRF) in a profile was calculated as a proportion of the total peak height of all TRFs [1]. Any peak with <0.5% of total fluorescence units was removed from the data before statistical analysis.

**Determination of microbial biomass**

Soil samples were fumigated with chloroform for 24 h and C was extracted by shaking 2 g or 7 g soil dry weight (for organic and mineral soils, respectively) in 40 ml 0.05 M K_2_SO_4_ for 30 min at 150 rpm and filtering through Fisherbrand FB59251 filter papers overnight in the cold room. DOC concentration was measured by UV absorbance spectrometry using a Trios ProPS (Zeiss, Germany), 10-mm path length and calculated based on the absorbance (190-360 nm) using MSDA_XE software [2].

**Sample preparation for pyrosequencing analysis**

One-way/unidirectional read amplicon sequencing was used to increase the number and length of unidirectional reads, providing better identification in complex samples. Products of triplicate PCR reactions products per sample were pooled to reduce reaction-level PCR-bias and purified with the Agencourt®AMPure®XP (Beckman Coulter, USA). Purified PCR products were then quantified using the Quant-iT™ PicoGreen® kit and pooled to create eight different libraries (3 for fungi and 5 for archaea and bacteria) in 10 µl, at a final concentration of 10 ng µl^-1^. Each library corresponded to one pyrosequencing region. All amplicons were sequenced using the 454 GS FLX Titanium platform at the Centre for Genomics Research, University of Liverpool, UK.

**Enzyme activity**

To determine the *β-*glucosidase activity, 0.1 g moist soil was placed in an Eppendorf tube and 0.025 ml toluene, 0.4 ml MUB buffer (pH 6.0) and 0.1 ml PNG-substrate were added. Autoclaved soil (20 min at 121 **°**C) was used as a control. After incubation for 1 h, 0.1 ml CaCl_2_ (0.5 M) and 0.4 ml Tris buffer pH 12 were added, tubes were shaken and centrifuged for 1 min at 3,000 rpm and colour intensity was measured spectrophotometrically at 400 nm.

To determine the dehydrogenase activity, 0.1 g soil was mixed with 0.15 ml Tris buffer and 0.2 ml INT solution, with autoclaved soil as control. Soil was extracted with 1 ml of acetone after incubation for 2 h at the temperatures described above, centrifuged and kept in the dark until reading colour intensity at 464 nm.

**Statistical analyses**

The fingerprint data were automatically reduced to single points, which were projected into a two-dimensional space (biplot) using canonical variate analysis (CVA). CVA was performed using 100 iterations with random starting configurations to ensure that minimum stress was achieved for the final analysis.

Barcode, linker primer and reverse primer sequences were removed from the raw sequence reads using the ‘split_libraries.py’ script while setting a minimum sequence length of 200 and a minimum quality score of 20. The Acacia (v1.52.b0) tool was used with default options to remove pyrosequencing noise [3]. Potential chimeras were removed using the UCHIME utility of the USEARCH v6.0.307 tool by aligning prokaryotic and fungal amplicons against the ‘Gold’ [4] and UNITE databases [5], respectively. A minimum of 2533 reads and 4062 reads were respectively obtained for the fungal or the archaeal-bacterial communities per sample. Then, all samples were rarefied to these common read counts before further analyses. Similar sequences were binned into OTUs using ‘UCLUST’ (minimum pairwise identity of 97%). In average, 722 (± 92) archaeal-bacterial OTUs and 478 (± 112) fungal OTUs were defined after two pyrosequencing runs.

Taxonomic assignments were made using the RDP classifier [6] trained on the Greengenes reference database sequences (gg_12_10) [7] for the prokaryotic OTUs, and based on BLAST [8] matching against the UNITE/QIIME ITS reference OTUs [5] for the fungal ITS sequences.

**References**

1. Singh BK, Nazaries L, Munro S, Anderson IC, Campbell CD. Use of Multiplex Terminal Restriction Fragment Length Polymorphism for rapid and simultaneous analysis of different components of the soil microbial community. Applied and Environmental Microbiology. 2006;72:7278-85.
2. Sandford R, Bol R, Worsfold P. In situ determination of dissolved organic carbon in freshwaters using a reagentless UV sensor. Journal of Environmental Monitoring 2010;12(9):1678-83.
3. Bragg L, Stone G, Imelfort M, Hugenholtz P, Tyson GW. Fast, accurate error-correction of amplicon pyrosequences using Acacia. Nat Methods. 2012;9(5):425-6.
4. Edgar R, Haas B, Clemente J, Quince C, Knight R. UCHIME improves sensitivity and speed of chimera detection. Bioinformatics. 2011;27(16):2194-200.
5. Abarenkov K, Henrik Nilsson R, Larsson KH, Alexander IJ, Eberhardt U, Erland S, et al. The UNITE database for molecular identification of fungi--recent updates and future perspectives. New Phytologist. 2010;186(2):281-5.
6. Wang Q, Garrity G, Tiedje J, Cole J. Naive Bayesian classifier for rapid assignment of rRNA sequences into the new bacterial taxonomy. Applied and environmental microbiology. 2007;73(16):5261-7.
7. McDonald D, Price M, Goodrich J, Nawrocki E, DeSantis T, Probst A, et al. An improved Greengenes taxonomy with explicit ranks for ecological and evolutionary analyses of bacteria and archaea. ISME journal. 2012;6(3):610–8.
8. Altschul SF, Madden TL, Schaffer AA, Zhang J, Miller W, Lipman DJ. Gapped BLAST and PSI-BLAST: a new generation of protein database search programs. Nucleic Acids Research. 1997;25:3389-402.
