# Supplementary material for: The Role of Microbial Community Composition in Controlling Soil Respiration Responses to Temperature
Source: PLoS One. 2016 Oct 31;11(10):e0165448. doi: 10.1371/journal.pone.0165448 (PMC5087920; doi:10.1371/journal.pone.0165448)
Supplement: S1 Table — (DOCX) [file pone.0165448.s008.docx]

S1 Table. List of 454 sequencing (Roche) adaptors used

| **MID ROCHE** | **Sequence** | **Microbial communities targeted** |
| --- | --- | --- |
| MID-1 | ACGAGTGCGT | Bacteria-Archaea and Fungi |
| MID-2 | ACGCTCGACA | Bacteria-Archaea and Fungi |
| MID-3 | AGACGCACTC | Bacteria-Archaea and Fungi |
| MID-4 | AGCACTGTAG | Bacteria-Archaea and Fungi |
| MID-5 | ATCAGACACG | Bacteria-Archaea and Fungi |
| MID-6 | ATATCGCGAG | Bacteria-Archaea and Fungi |
| MID-7 | CGTGTCTCTA | Bacteria-Archaea and Fungi |
| MID-8 | CTCGCGTGTC | Bacteria-Archaea and Fungi |
| MID-10 | TCTCTATGCG | Bacteria-Archaea and Fungi |
| MID-11 | TGATACGTCT | Bacteria-Archaea and Fungi |
| MID-13 | CATAGTAGTG | Bacteria-Archaea and Fungi |
| MID-14 | CGAGAGATAC | Bacteria-Archaea and Fungi |
| MID-15 | ATACGACGTA | Bacteria-Archaea and Fungi |
| MID-16 | TCACGTACTA | Bacteria-Archaea and Fungi |
| MID-17 | CGTCTAGTAC | Bacteria-Archaea and Fungi |
| MID-18 | TCTACGTAGC | Bacteria-Archaea and Fungi |
| MID-19 | TGTACTACTC | Fungi |
| MID-20 | ACGACTACAG | Fungi |
| MID-21 | CGTAGACTAG | Fungi |
| MID-22 | TACGAGTATG | Fungi |
| MID-23 | TACTCTCGTG | Fungi |
| MID-24 | TAGAGACGAG | Fungi |
| MID-25 | TCGTCGCTCG | Fungi |
| MID-27 | ACGCGAGTAT | Fungi |
| MID-28 | ACTACTATGT | Fungi |
| MID-30 | AGACTATACT | Fungi |
