# Supplementary material for: The Role of Microbial Community Composition in Controlling Soil Respiration Responses to Temperature
Source: PLoS One. 2016 Oct 31;11(10):e0165448. doi: 10.1371/journal.pone.0165448 (PMC5087920; doi:10.1371/journal.pone.0165448)
Supplement: S2 Table — * Spearman correlation results differ at °P<0.1, or *P<0.05. a The number of soils tested is n = 8. 1 Values obtained from bulk soils initially collected. 2 Values based on the ratio between control and cooled treatments. (DOCX) [file pone.0165448.s009.docx]

S2 Table. Spearman correlation results.

| **Factors** | **RR_MT_ (control/cooled treatments)**^a^ | |
| --- | --- | --- |
|  | **F** | ***P*** |
| ^1^MAT | -0.38* | 0.05 |
| ^1^% C | 0.24 | 0.23 |
| ^1^% N | 0.06 | 0.75 |
| ^1^C:N | 0.23 | 0.25 |
| ^1^pH | -0.24 | 0.22 |
| ^2^QPCR Bacteria | 0.02 | 0.90 |
| ^2^QPCR Fungi | -0.01  0.03 | 0.99 |
| ^2^Archaea | 0.44 | 0.17 |
| ^2^*Acidobacteria* | 0.39 | 0.23 |
| ^2^*Actinobacteria* | -0.53° | 0.08 |
| ^2^*Armatimonadetes* | 0.00 | 1.00 |
| ^2^*Bacteroidetes* | -0.49 | 0.12 |
| ^2^*Chloroflexi* | 0.43 | 0.18 |
| ^2^*Firmicutes* | 0.04 | 0.89 |
| ^2^*Gemmatimonadetes* | -0.18 | 0.59 |
| ^2^*Nitrospirae*  *2* | 0.46 | 0.18 |
| ^2^*Planctomycetes* | 0.51 | 0.10 |
| ^2^*Proteobacteria* | -0.04 | 0.89 |
| ^2^*TM6* | -0.15 | 0.66 |
| ^2^*Verrucomicrobia* | -0.47 | 0.14 |
| ^2^*Ascomycota* | -0.01 | 0.98 |
| ^2^*Basidiomycota* | -0.71* | 0.01 |
| ^2^Others | 0.41 | 0.20 |

* Spearman correlation results differ at °*P*<0.1, or **P*˂0.05.

^a^ The number of soil tested is *n*=8.

^1^ Values obtained from bulk soils initially collected. ^2^ Values based on the ratio between control and cooled treatments.
