# Supplementary material for: The Role of Microbial Community Composition in Controlling Soil Respiration Responses to Temperature
Source: PLoS One. 2016 Oct 31;11(10):e0165448. doi: 10.1371/journal.pone.0165448 (PMC5087920; doi:10.1371/journal.pone.0165448)
Supplement: S3 Table — D.L: Below the detection limit; Underlined phyla represent fungal phyla; aMBGA: Marine Benthic Group A of Archaea. * indicates that higher OTU abundance was significantly different between treatments, confirmed by Bonferroni and FDR tests (P<0.05). pc: precooling. (DOCX) [file pone.0165448.s010.docx]

S3 Table. Significantly different OTUs (control / cooled treatment) determined by pyrosequencing.

| **Soil** | **Treatment comparison** | **Relative abundance (mean %)** | | | | **Phylum** | **Consensus Lineage (phylogenetic classification)** |
| --- | --- | --- | --- | --- | --- | --- | --- |
|  |  | **precooling** | | **control** | **Cooled** |  |  |
| 1H | pc/cooled | D.L. | D.L. | | 2.18* | *Ascomycota* | *Lecanorales* (order) |
| 1H | pc/cooled | D.L. | 0.62 | | 0.72* | *Ascomycota* | *Neonectria* (genus) |
| 1H | pc/cooled | D.L. | D.L. | | 0.60* | *Basidiomycota* | *Hyphoderma* (genus) |
| 1H | pc/cooled | 1.25 | 2.56 | | 2.59* | *Acidobacteria* | Candidatus *Solibacter* (genus) |
| 1H | pc/control | 1.10* | 0.68 | | 0.83 | *Planctomycetes* | *Pirellulaceae* (family) |
| 1H | pc/control | D.L. | 0.40* | | D.L. | *Elusimicrobia* | *Elusimicrobiales* (order) |
| 1H | control/cooled | 1.47 | 1.42* | | D.L. | *Crenarchaeota* | MBGA^a^ (class) |
| 1H | control/cooled | 1.91 | 2.38* | | 0.58 | Bacteria | AD3 other |
| 1H | control/cooled | 0.25 | 0.32 | | 0.53* | *Verrucomicrobia* | *Opitutae* (class) |
| 1H | control/cooled | 13.77 | 12.98* | | 3.45 | *Chloroflexi* | *Thermogemmatisporaceae* (family) |
| 1H | control/cooled | D.L. | D.L. | | 2.18* | *Ascomycota* | *Lecanorales* (order) |
| 1H | control/cooled | 1.25 | 0.61 | | 2.50* | *Basidiomycota* | *Sebacinales* (order) |
| 1H | control/cooled | D.L. | D.L. | | 0.60* | *Basidiomycota* | *Hyphoderma* (genus) |
| 1H | control/cooled | 0.54 | 1.98* | | D.L. | *Ascomycota* | *Monographella* (genus) |
| 1H | control/cooled | 1.16 | 2.84* | | 0.31 | *Ascomycota* | *Archaeorhizomyces* (genus) |
| 1H | control/cooled | 7.93 | 4.09 | | 11.61* | *Basidiomycota* | *Cortinariaceae* (family) |
| 1H | control/cooled | 0.28 | 0.80* | | D.L. | *Ascomycota* | *Trichoglossum* (genus) |
| 2G | pc/cooled | D.L. | D.L. | | 0.50* | *Proteobacteria* | Ellin329 (order) |
| 2G | pc/cooled | 1.81* | 0.81 | | D.L. | *Basidiomycota* | *Cortinariaceae* (family) |
| 2G | control/cooled | 7.09 | 9.27* | | 6.69 | *Ascomycota* | *Archaeorhizomyces* (genus) |
| 3A | pc/cooled | 0.76 | 0.74 | | 1.04* | *Actinobacteria* | *Solirubrobacterales* (order) |
| 3A | control/cooled | 8.88 | 7.83* | | 7.29 | *Acidobacteria* | subdivision 6 (class) |
| 3A | control/cooled | 1.28 | 1.22 | | 1.45* | *Actinobacteria* | *Gaiellaceae* (family) |
| 3A | control/cooled | 0.82 | 0.80 | | 0.87* | *Planctomycetes* | *Gemmataceae* (family) |
| 3C | pc/control | 1.77* | 1.58 | | 1.88 | *Proteobacteria* | *Rhodospirillaceae* (family) |
| 3C | pc/control | 0.94* | 0.42 | | 0.70 | *Planctomycetes* | *Gemmataceae* (family) |
| 3C | control/cooled | 4.34 | 2.81 | | 4.68* | *Planctomycetes* | *Phycisphaerae* (class) |
| 3C | control/cooled | 18.71 | 21.74* | | 18.58 | *Actinobacteria* | *Actinomycetales* (order) |
| 4A | pc/cooled | 0.47* | D.L. | | D.L. | *Chloroflexi* | Dolo-23 (family) |
| 4A | pc/cooled | 0.73* | 0.59 | | 0.56 | *Planctomycetes* | *Planctomyces* (genus) |
| 4A | pc/cooled | 5.13 | 5.82 | | 6.19* | *Acidobacteria* | *Chloracidobacteria* (class) |
| 4A | pc/cooled | 0.30 | 0.36 | | 0.47* | *Proteobacteria* | *Myxococcales* (order) |
| 4A | pc/cooled | 5.01* | 4.57 | | 3.60 | *Planctomycetes* | *Pirellulaceae* (family) |
| 4A | control/cooled | 0.45 | 0.30* | | 0.26 | *Planctomycetes* | CL500-15 (order) |
| 4C | pc/cooled | 2.92* | 2.40 | | 2.70 | *Planctomycetes* | *Isosphaeraceae* (family) |
| 4C | pc/cooled | 0.55* | 0.41 | | 0.48 | *Acidobacteria* | DS-18 (order) |
| 4C | pc/cooled | 9.37* | 8.17 | | 7.48 | *Planctomycetes* | *Phycisphaerae* (class) |
| 4C | pc/cooled | 1.49* | 0.62 | | 0.89 | *Planctomycetes* | *Gemmataceae* (family) |
| 4C | pc/control | D.L. | 0.45* | | D.L. | Bacteria GN02 | GKS2-174 (class) |
| 4C | pc/control | 0.42 | 0.55* | | 0.37 | *Planctomycetes* | *Planctomyces* (genus) |
| 4C | control/cooled | D.L. | 0.45* | | D.L. | Bacteria GN02 | GKS2-174 (class) |
| 4D | pc/cooled | 1.06 | 0.87 | | 1.14* | *Basidiomycota* | *Inocybe* (genus) |
| 4D | pc/control | 0.60* | 0.57 | | 0.58 | *Ascomycota* | *Penicillium* (genus) |
| 4D | pc/control | 1.06* | 0.87 | | 1.14 | *Basidiomycota* | *Inocybe* (genus) |
|  |  |  |  | |  |  |  |

D.L: Below the detection limit; Underlined phyla represent fungal phyla; ^a^MBGA: Marine Benthic Group A of Archaea

* indicates that higher OTU abundance was significantly different between treatments, confirmed by Bonferroni and FDR tests (*P*˂0.05). pc: precooling.
