# Supplementary material for: The Role of Microbial Community Composition in Controlling Soil Respiration Responses to Temperature
Source: PLoS One. 2016 Oct 31;11(10):e0165448. doi: 10.1371/journal.pone.0165448 (PMC5087920; doi:10.1371/journal.pone.0165448)
Supplement: S4 Table — Temperature range used for determining Q10 of enzyme activity was 1 to 29°C for soils 1A to 2H, 1 to 19°C for 3G soil, 1 to 13°C for 3H soil, and 1 to 32°C for soils 4A to 4H. These different temperature ranges were selected to reflect seasonal temperature changes in soils depending of the site of origin. (DOCX) [file pone.0165448.s011.docx]

S4 Table. Temperature sensitivity of *β-*glucosidase activity expressed per chloroform fumigation extraction biomass (µg PNP mg^-1^ CFE-flush h^-1^).

| **Soil** | **Treatment** | **Parameter** | **95% confidence intervals** | | **Parameter** | **95% confidence intervals** | |
| --- | --- | --- | --- | --- | --- | --- | --- |
|  |  | ***a*** | **lower** | **upper** | ***Q*_10_** | **lower** | **upper** |
| 1A | control | 55.87 | 46.48 | 67.15 | 2.27 | 2.05 | 2.51 |
|  | cooled | 69.48 | 61.50 | 78.49 | 2.01 | 1.90 | 2.16 |
| 1C | control | 1751.10 | 1456.80 | 2104.85 | 1.40 | 1.27 | 1.55 |
|  | cooled | 2145.23 | 1822.57 | 2525.01 | 1.35 | 1.23 | 1.46 |
| 1D | control | 266.93 | 211.86 | 336.30 | 1.63 | 1.45 | 1.86 |
|  | cooled | 358.53 | 272.05 | 472.48 | 1.48 | 1.27 | 1.70 |
| 1G | control | 85.97 | 71.16 | 103.96 | 1.79 | 1.62 | 1.97 |
|  | cooled | 84.02 | 73.11 | 96.54 | 1.79 | 1.65 | 1.92 |
| 1H | control | 120.18 | 102.21 | 141.32 | 1.86 | 1.70 | 2.01 |
|  | cooled | 170.55 | 139.07 | 209.14 | 1.70 | 1.52 | 1.90 |
| 2C | control | 111.27 | 89.21 | 138.80 | 1.79 | 1.58 | 1.99 |
|  | cooled | 143.88 | 119.46 | 173.47 | 1.68 | 1.52 | 1.88 |
| 2D | control | 115.01 | 79.28 | 166.67 | 1.97 | 1.45 | 2.69 |
|  | cooled | 97.03 | 48.57 | 194.03 | 1.82 | 1.02 | 3.22 |
| 2G | control | 87.53 | 68.72 | 111.39 | 1.92 | 1.68 | 2.18 |
|  | cooled | 88.32 | 76.17 | 102.51 | 1.93 | 1.79 | 2.10 |
| 2H | control | 185.68 | 136.46 | 252.40 | 1.65 | 1.40 | 1.95 |
|  | cooled | 164.19 | 140.47 | 192.10 | 1.75 | 1.60 | 1.90 |
| 3C | control | 148.56 | 118.75 | 185.86 | 1.86 | 1.54 | 2.25 |
|  | cooled | 104.27 | 57.69 | 188.29 | 2.46 | 1.51 | 4.01 |
| 3G | control | 26.68 | 19.53 | 36.49 | 2.14 | 1.65 | 2.77 |
|  | cooled | 23.10 | 19.89 | 26.84 | 2.20 | 1.93 | 2.48 |
| 3H | control | 180.55 | 141.74 | 229.98 | 1.65 | 1.23 | 2.18 |
|  | cooled | 179.65 | 132.42 | 243.96 | 1.68 | 1.17 | 2.39 |
| 4A | control | 324.08 | 278.11 | 377.66 | 1.54 | 1.42 | 1.65 |
|  | cooled | 327.34 | 281.74 | 380.32 | 1.57 | 1.46 | 1.68 |
| 4C | control | 132.29 | 96.25 | 181.64 | 1.88 | 1.62 | 2.18 |
|  | cooled | 108.85 | 87.71 | 135.10 | 1.88 | 1.70 | 2.08 |
| 4D | control | 44.70 | 36.86 | 54.22 | 1.93 | 1.77 | 2.12 |
|  | cooled | 41.39 | 35.77 | 47.85 | 1.97 | 1.84 | 2.12 |
| 4G | control | 158.86 | 137.69 | 183.28 | 1.72 | 1.60 | 1.84 |
|  | cooled | 168.17 | 145.18 | 194.61 | 1.70 | 1.58 | 1.84 |
| 4H | control | 111.83 | 90.47 | 138.24 | 1.68 | 1.51 | 1.86 |
|  | cooled | 92.67 | 70.46 | 121.7537 | 1.70 | 1.48 | 1.93 |

Temperature range used for determining *Q*_10_ of enzyme activity was 1 to 29 °C for soils 1A to 2H, 1 to 19 °C for 3G soil, 1 to 13 °C for 3H soil, and 1 to 32 °C for soils 4A to 4H.
