# Supplementary material for: The Role of Microbial Community Composition in Controlling Soil Respiration Responses to Temperature
Source: PLoS One. 2016 Oct 31;11(10):e0165448. doi: 10.1371/journal.pone.0165448 (PMC5087920; doi:10.1371/journal.pone.0165448)
Supplement: S5 Table — *Q10 values differ at the 0.05 level (95% confidence intervals do not overlap). (DOCX) [file pone.0165448.s012.docx]

S5 Table. Temperature sensitivity of dehydrogenase activity expressed per chloroform fumigation extraction biomass (µg INF mg^-1^ CFE-flush h^-1^).

| **Soil** | **Treatment** | **Parameter** | **95% confidence intervals** | | **Parameter** | **95% confidence intervals** | |
| --- | --- | --- | --- | --- | --- | --- | --- |
|  |  | **a** | **lower** | **upper** | ***Q*_10_** | **lower** | **upper** |
| 1A | *control* | 4.68 | 2.42 | 9.05 | 1.99 | 1.40 | 2.80 |
|  | *cooled* | 5.18 | 2.80 | 9.56 | 1.95 | 1.46 | 2.64 |
|  |  |  |  |  |  |  |  |
| 1G | *control* | 4.80 | 2.87 | 8.01 | 2.20 | 1.68 | 2.92 |
|  | *cooled* | 3.68 | 2.06 | 6.57 | 2.34 | 1.72 | 3.22 |
|  |  |  |  |  |  |  |  |
| 1H | *control* | 41.76 | 31.28 | 55.81 | 1.90 | 1.62 | 2.23 |
|  | *cooled* | 40.61 | 27.47 | 60.04 | 2.18 | 1.77 | 2.69 |
|  |  |  |  |  |  |  |  |
| 2C | *control* | 80.40 | 64.85 | 99.68 | 1.31 | 1.17 | 1.48 |
|  | *cooled* | 87.18 | 70.95 | 107.02 | 1.32 | 1.19 | 1.48 |
|  |  |  |  |  |  |  |  |
| 2D | *control* | 10.97 | 4.83 | 24.93 | 3.10 | 1.46 | 6.55 |
|  | *cooled* | 15.13 | 7.43 | 30.85 | 2.41 | 1.26 | 4.62 |
|  |  |  |  |  |  |  |  |
| 2G | *control* | 46.90 | 39.10 | 56.20 | 1.55* | 1.42 | 1.72 |
|  | *cooled* | 39.13 | 33.38 | 45.88 | 1.88* | 1.73 | 2.05 |
|  |  |  |  |  |  |  |  |
| 2H | *control* | 65.30 | 45.02 | 94.63 | 1.82 | 1.49 | 2.25 |
|  | *cooled* | 95.87 | 77.87 | 117.92 | 1.54 | 1.38 | 1.73 |
|  |  |  |  |  |  |  |  |
| 3G | *control* | 14.30 | 10.74 | 19.03 | 2.39 | 1.88 | 3.03 |
|  | *cooled* | 15.47 | 13.08 | 18.28 | 2.14 | 1.86 | 2.46 |
|  |  |  |  |  |  |  |  |
| 3H | *control* | 83.26 | 67.56 | 102.62 | 1.35 | 1.14 | 1.60 |
|  | *cooled* | 80.48 | 68.17 | 95.01 | 1.42 | 1.23 | 1.63 |
|  |  |  |  |  |  |  |  |
| 4A | *control* | 26.60 | 20.84 | 33.95 | 2.14 | 1.90 | 2.41 |
|  | *cooled* | 31.63 | 26.15 | 38.24 | 2.05 | 1.88 | 2.25 |
|  |  |  |  |  |  |  |  |
| 4C | *control* | 33.08 | 26.60 | 41.18 | 2.12 | 1.90 | 2.34 |
|  | *cooled* | 26.74 | 18.34 | 38.98 | 2.12 | 1.77 | 2.56 |
|  |  |  |  |  |  |  |  |
| 4D | *control* | 27.22 | 23.97 | 30.91 | 2.05 | 1.93 | 2.18 |
|  | *cooled* | 29.78 | 23.45 | 37.83 | 1.95 | 1.73 | 2.20 |
|  |  |  |  |  |  |  |  |
| 4G | *control* | 33.21 | 25.95 | 42.52 | 1.79 | 1.62 | 1.99 |
|  | *cooled* | 28.45 | 22.58 | 35.84 | 1.90 | 1.72 | 2.08 |

**Q*_10_ values differ at the 0.05 level (95% confidence intervals do not overlap)
